# Consumption of Nuts and Seeds and Health Outcomes Including Cardiovascular Disease, Diabetes and Metabolic Disease, Cancer, and Mortality: An Umbrella Review

**DOI:** 10.1093/advances/nmac077

**Published:** 2022-08-30

**Authors:** Rajiv Balakrishna, Tonje Bjørnerud, Mitra Bemanian, Dagfinn Aune, Lars T Fadnes

**Affiliations:** Department of Global Public Health and Primary Care, University of Bergen, Bergen, Norway; Department of Global Public Health and Primary Care, University of Bergen, Bergen, Norway; Department of Global Public Health and Primary Care, University of Bergen, Bergen, Norway; Department of Addiction Medicine, Haukeland University Hospital, Bergen, Norway; Department of Epidemiology and Biostatistics, School of Public Health, Imperial College London, London, United Kingdom; Department of Nutrition, Oslo New University College, Oslo, Norway; Department of Endocrinology, Morbid Obesity and Preventive Medicine, Oslo University Hospital, Oslo, Norway; Unit of Cardiovascular and Nutritional Epidemiology, Institute of Environmental Medicine, Karolinska Institute, Stockholm, Sweden; Department of Global Public Health and Primary Care, University of Bergen, Bergen, Norway; Department of Addiction Medicine, Haukeland University Hospital, Bergen, Norway

**Keywords:** nuts, cardiovascular diseases, neoplasms, type 2 diabetes mellitus, diet, food, nutrition, mortality, biomarkers

## Abstract

Consumption of nuts and seeds is associated with a range of health outcomes. Summarizing the best evidence on essential health outcomes from the consumption of nuts is essential to provide optimal recommendations. Our objective is to comprehensively assess health outcome associations related to the consumption of nuts and seeds, using a culinary definition including tree nuts and peanuts (registered in PROSPERO: CRD42021258300). Health outcomes of interest include cardiovascular disease, cancer, diabetes, obesity, respiratory disease, mortality, and their disease biomarkers. We present associations for high compared with low consumption, per serving, and dose–response relations. MEDLINE, Embase, Cochrane, and Epistemonikos were searched and screened for systematic reviews and meta-analyses. Evidence was extracted from 89 articles on the consumption of nuts and relevant health outcomes, including 23 articles with meta-analysis on disease and mortality, 66 articles on biomarkers for disease, and 9 articles on allergy/adverse outcomes. Intake of nuts was associated with reduced risk of cardiovascular diseases and related risk factors, with moderate quality of evidence. An intake of 28 g/d nuts compared with not eating nuts was associated with a 21% RR reduction of cardiovascular disease (including coronary heart disease incidence and mortality, atrial fibrillation, and stroke mortality), an 11% risk reduction of cancer deaths, and 22% reduction in all-cause mortality. Nut consumption was also inversely associated with mortality from respiratory diseases, infectious diseases, and diabetes; however, associations between nut consumption and diabetes incidence were mixed. Meta-analyses of trials on biomarkers for disease generally mirrored meta-analyses from observational studies on cardiovascular disease, cancers, and diabetes. Allergy and related adverse reactions to nuts were observed in 1–2% of adult populations, with substantial heterogeneity between studies. Overall, the current evidence supports dietary recommendations to consume a handful of nuts and seeds per day for people without allergies to these foods.

## Teaser

**Statement of Significance:** This umbrella review provides comprehensive and up-to-date evidence on nut consumption and the risk of cardiovascular disease, cancers, diabetes, and mortality. It also presents per serving and dose–response relations, and evidence on biomarkers for diseases.

## Introduction

Nuts and seeds have been part of diets worldwide for millennia (1). Nuts and seeds are highly nutrient-dense dietary components, rich in macronutrients including MUFAs and PUFAs, proteins, and fibers (2, 3). They are also rich in vitamins and minerals, and a range of active metabolites such as phenolic acids, phytosterols, carotenoids, and polyphenolic compounds (2, 4–6). Some of the compounds present in nuts, including polyphenols, have been found to have antioxidant, antimicrobial, and antiproliferative properties (4, 7, 8). Nuts were utilized in ancient medicinal traditions, an example being Hippocrates’ description of almonds as a treatment for colds and coughs (1).

Nuts are botanically categorized as tree nuts and peanuts. Nuts have hard shells covering the seed, and examples of frequently consumed tree nuts include almonds, walnuts, hazelnuts, cashews, Brazil nuts, macadamias, and pistachios. Tree nuts and peanuts have many compositional/nutritional similarities, and even though peanuts are botanically classified as legumes, their culinary use is similar to tree nuts (9). Further, seeds such as sesame and sunflower are related food groups (10). Consumption of nuts and seeds varies between cultural settings, both in preferences for nut and seed types and the amounts consumed (11, 12), with higher consumption generally reported in Canada, some African countries, parts of Europe, and the Middle East, and lower intakes in South America.

Consumption of nuts and seeds has been inversely associated with the risks of cardiovascular disease, cancers, and respiratory diseases (13–18). Cardiovascular disease, cancer, respiratory diseases, diabetes, and neurodegenerative diseases are globally among the leading causes of death and life years lost (19–21), contributing to 32%/20%, 16%/13%, 11%/10%, 3%/2%, 5%/2%, and 2%/2% of deaths/life years lost from these outcomes, respectively. On the other hand, nut allergies and related reactions are potential unintended effects (22). Some compounds, such as phytates, might also reduce the bioavailability of some nutrients in the gastrointestinal tract (23). Nuts might also impact the microbiota, but the results are uncertain regarding whether they tend to have more prebiotic properties and stimulate the growth of nonpathogenic gut bacteria, or promote pathogenic bacteria (24, 25). To contribute to optimizing intake levels through diet recommendations, both positive and adverse effects need to be considered. Therefore, summarizing the best evidence on health outcomes from consumption of nuts and seeds is essential. Umbrella reviews have been conducted focusing on cardiovascular and metabolic outcomes (26–28). However, these do not cover all relevant morbidities, and many relevant meta-analyses have been published subsequently. Thus, a comprehensive update could give more precise estimates and balance various health outcomes.

This umbrella review provides a systematic and comprehensive overview of the evidence on the consumption of nuts and seeds and the associations with various diseases, including high compared with low consumption, per serving, and dose–response relations. We have used a culinary definition of nuts and seeds, thus including tree nuts, peanuts, and seeds, and presenting data on biomarkers for diseases as intermediate causal factors contributing to understanding the evidence.

## Methods

To summarize the evidence from meta-analyses and systematic reviews on the consumption of nuts and relevant health outcomes such as cardiovascular disease, cancer, diabetes, obesity, respiratory disease, mortality, and their intermediate factors, we used an umbrella review framework (29, 30). The protocol for the study has been registered in PROSPERO (https://www.crd.york.ac.uk/prospero/display_record.php?ID=CRD42021258300).

### Eligibility criteria

We evaluated meta-analyses and systematic reviews presenting analyses from cohorts and trials on the consumption of nuts and seeds and associations with incidence and mortality of different diseases and intermediary factors related to these diseases. For inclusion and exclusion criteria, see below. Studies with a cross-sectional design or only presenting regional estimates not representative of a general population were excluded. No search restrictions were imposed on the publication date or publication status. We excluded articles written in languages other than English, German, French, Norwegian, Danish, or Swedish.

#### Inclusion criteria

We included meta-analyses and systematic reviews presenting analyses from longitudinal observation studies (e.g., cohorts, nested case-control) and trials, in which the exposure was consumption of nuts and seeds (using a culinary definition). The comparators were high compared with low consumption, per serving, and dose–response relation between exposure and outcomes. The outcomes were cardiovascular diseases, cancer, diabetes and metabolic disease, respiratory, infectious, and other diseases, adverse effects including allergies, and mortality, as well as intermediary factors for these diseases. Included articles were published in English, German, French, Norwegian, Danish, or Swedish, and indexed in MEDLINE, Embase, Cochrane, and Epistemonikos from inception to May 27, 2021.

#### Exclusion criteria

We excluded nonsystematic reviews and studies not presenting results for nuts separately but only as part of a combined diet.

### Types of outcome measures

Outcomes included were the following: coronary heart disease, coronary heart disease mortality, cardiovascular disease, cardiovascular disease mortality, cancer mortality, diabetes mellitus, diabetes mortality, obesity or overweight, metabolic syndrome, heart failure, stroke and subtypes including hemorrhagic stroke incidence, ischemic stroke incidence, stroke mortality, infectious disease and related mortality, kidney disease and related mortality, neuro-degenerative disease mortality, respiratory disease mortality, adverse effects including allergies and anaphylactic reactions, and all-cause mortality (**Supplemental Table 1**). We also assessed biomarkers for disease (intermediate factors), including blood lipids, cholesterols, endothelial function, blood pressure, body composition and weight, hunger and fullness, glucose and insulin, inflammation, and gut microbiota.

### Information sources

**FIGURE 1:**
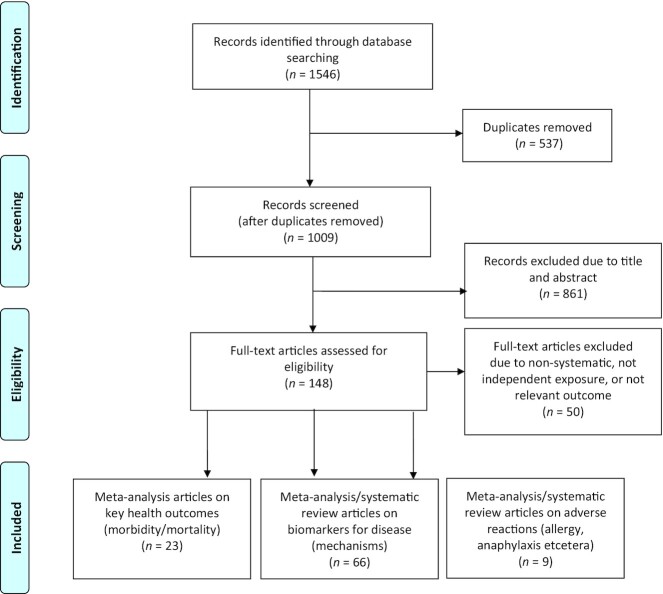
Study selection for the umbrella analysis of health outcomes of nuts and seeds.

Overall, in collaboration with an experienced librarian, 1546 records were retrieved from the databases in MEDLINE, Embase, Cochrane, and Epistemonikos (also extracting through CINAHL, PsycINFO, LILACS, DARE, The Campbell Collaboration online library, JBI Database, and EPPI-Centre Evidence Library). The search period was from inception to May 27, 2021. After automatic deduplication in EndNote, 1009 records remained (see **Figure 1** and details of search in **Supplemental Material**). No limits were applied for language or publication date. This systematic review has made efforts to adhere to the Preferred Reporting Items for Systematic Reviews and Meta-Analyses (PRISMA) criteria (31).

### Search

The search included the following terms (≥1 of *1*, *2*, and *3*): *1*) nut, almond, Brazil nut, cashew nut, hazelnut, pecan, pistachio, walnut, peanut, macadamia, sesame, oilseed, hickory, seeds, pine seed, sunflower seed, chia, poppy seed, hemp seed, quinoa, pumpkin seed, or flaxseed; *2*) intake, consumption, eat, or diet; and *3*) systematic review or meta-analysis.

For further details on search, see Supplemental Material. The references were imported into EndNote X9.

### Study selection

The 2 first authors (RB and TB) screened the imported references. The screening was done by reading the title, abstract, and assessing full-text articles assumed to be relevant. Two authors read all obtainable relevant articles in full text, and possible differences in assessment were discussed between the authors and resolved by consensus. None of the available meta-analyses reported on total cancer incidence, although some meta-analyses combined different cancer types in an overall analysis (32–35). None of the original studies included in these meta-analyses reported on total cancer incidence, only on the incidence of specific cancers, and because of this the results for different cancers combined were not considered reliable and were not used in the current analysis. Data values extracted were also double-checked.

### Data collection process and data items

Data considered relevant were extracted into a Microsoft Excel table, and information was gathered on the first author, title, primary outcome(s), aims of the studies, conclusion, exposure (types of nuts/seeds), inclusion and exclusion criteria, design, type and number of studies, number of participants, number of cases/outcomes, outcome measures, heterogeneity, findings reported on high vs. low intake, findings on dose-response or per serving, and findings categorized otherwise (Supplemental Table 1). We used the data from the source published last for duplicate data identified. A total of 148 full-text articles were assessed in detail.

### Risk of bias in individual studies and across studies

The risk of bias was assessed with the AMSTAR-2 tool [A MeaSurement Tool to Assess systematic Reviews (version 2)] (36). The quality of the reviews was categorized into high/moderate/low (e.g., AMSTAR-2: high). Details from the assessments are listed in **Supplemental Table 2**.

### Analysis

Tables with extracted data from included studies were made. These data were summarized in figures visualizing the associations between nuts and various health outcomes for low compared with high, per serving, and dose–response. For per serving, when units other than per serving of 20–30 g/d were used, a conversion of the RR was estimated (for 4 servings of 28 g/wk: RR^(7/4)^; for 1 serving per week: RR^4^; for servings of 20 g/d: RR^1^; for servings of 12 g/d: RR^2^). We present forest plots for the most comprehensive meta-analysis for each outcome measure (and similar emphasizing the most recent meta-analyses). The most comprehensive was defined as the relevant meta-analysis including the most relevant studies and having the most participants with the relevant outcomes. The forest plots include information on source/reference, the number of participants and cases, included studies, and heterogeneity. We also present data for nuts and subgroups of nuts and groups of outcomes, including:

1. Cardiovascular disease/coronary heart disease/stroke/heart failure/atrial fibrillation
2. All-cause mortality
3. Diabetes, diabetes mortality, and metabolic syndrome
4. Cancer mortality
5. Other morbidities including infectious disease mortality, kidney disease mortality, neurodegenerative disease mortality, respiratory disease mortality.

For dose–response, we present relevant data from meta-analyses, extracted values through the Web Plot Digitizer tool (https://apps.automeris.io/wpd/), and present these in the form of supplemental figures. Stata SE 17 (StataCorp LLC) was used for data analysis and graphical presentation.

## Results

Twenty-three meta-analysis articles provided 190 outcome measures for disease and disease-related mortality (Supplemental Tables 1 and **3**) (13–16, 32–35, 37–52). Most outcome measures were available for all-cause mortality, cancer mortality, and cardiovascular disease, with subcategories including related incidence and mortality, coronary heart disease, and stroke. Sixty-six full-text articles provided data on biomarkers for disease and disease mechanisms (53–118), and most of these were trials.

### Cardiovascular disease

**FIGURE 2:**
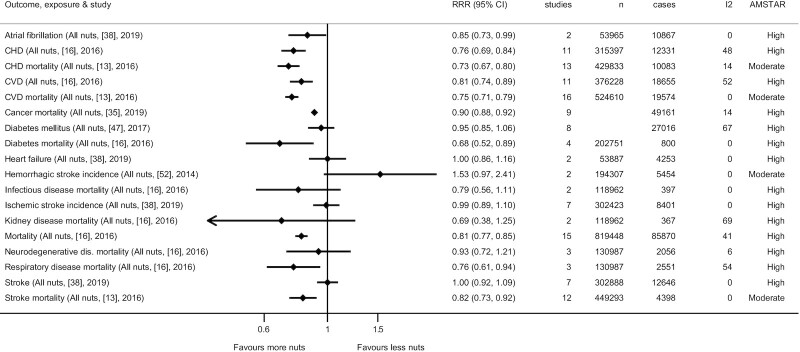
Summary of associations from the most comprehensive meta-analyses between high compared with low consumption of nuts and risk of various morbidities and mortalities. Reference number is listed in brackets and search year is listed within the parentheses.

**FIGURE 3:**
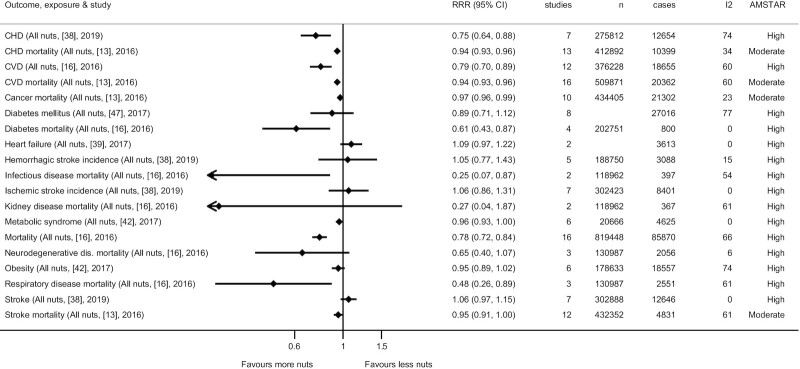
Summary of per serving associations from the most comprehensive meta-analyses between consumption of 28 g/d nuts and risk of various morbidities and mortalities. Reference number is listed in brackets and search year is listed within the parentheses.

**FIGURE 4:**
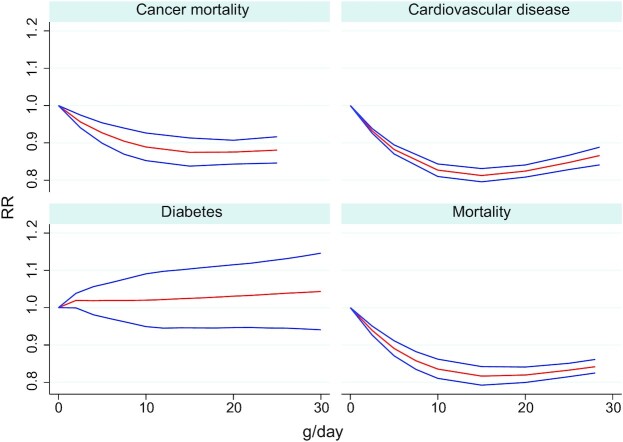
Summary of dose–response associations from the most comprehensive meta-analyses between consumption of nuts and risk of cancer mortality, cardiovascular disease, diabetes, and all-cause mortality. Red lines indicate the RR associations estimate whereas blue lines represent the CI of the RRs.

Meta-analyses indicate inverse associations between high compared with low consumption of nuts and cardiovascular diseases (**Supplemental Figures 1–4**; **Figure 2**) (16, 37–39, 43–45, 48–50, 52). Similar findings are also seen for coronary heart disease and related mortality. For per serving data, a daily intake of 28 g nuts is associated with an RR of 0.79 (95% CI: 0.70–0.89; *I*^2^: 60%; AMSTAR-2: high) for overall cardiovascular disease, 0.75 (95% CI: 0.64–0.88; *I*^2^: 74%; AMSTAR-2: high) for coronary heart disease, and 0.78 (95% CI: 0.73–0.83; *I*^2^: 60%; AMSTAR-2: moderate) for cardiovascular mortality (**Figure 3**). The associations were generally similar between the intake of tree nuts compared with peanuts and cardiovascular outcomes. An inverse association was observed between intake of peanuts and stroke, although this was nonsignificant for tree nuts. Dose–response associations between nut consumption and risk of cardiovascular diseases and coronary heart diseases suggest optimal intake levels of ∼15–20 g/d nuts and limited benefits in increasing intake beyond 1 serving of 28 g/d (**Figure 4**; **Supplemental Figures 5–7**). There were 376,228 participants and 18,655 cases in the overall cardiovascular disease analyses for all nuts.

#### Mechanisms related to cardiovascular disease

Meta-analyses investigating the effects of nut and seed consumption on biomarkers for disease (intermediary outcomes) related to cardiovascular disease generally reported favorable effects on blood lipid profile (linked with reduced risk of diseases), particularly for total cholesterol, LDL, triglycerides, and apoB (56, 59, 62, 69–71, 75, 77, 79, 81, 85, 93, 99–101, 105) (**Table 1**). In contrast, some reported no significant biomarker change (66, 72, 73, 77, 81, 90, 109). Evidence on vascular endothelial function and blood pressure outcomes either indicated favorable (55, 63, 65, 66, 73, 77, 78, 86, 95, 108, 112, 116), or neutral/no significant biomarker change (55, 56, 62, 63, 65, 66, 69, 71–73, 79, 80, 85, 89, 109). However, the duration of many of these trials could have been too short to identify potential effects on changes in blood pressure and hypertension (60, 62, 63, 72, 73, 80, 89). Meta-analyses of observational studies reported an inverse association between consumption of nuts and risk of hypertension (41, 107, 118).

**TABLE 1:** List of biomarkers for various disease and intermediate mechanisms for various morbidities from systematic reviews and meta-analyses including cardiovascular, diabetes and weight, and other outcomes^1^

	Favorable	Neutral	Unfavorable
*Blood lipids*			
HDLs	(72)	(59), (85), (62), (69), (70), (71), (73), (77), (81), (90), (93), (99), (100), (101), (105), (66), (75), (109), (56)	(79)
LDLs	(62), (69), (70), (81), (93), (99), (100), (105), (75), (79), (56)	(71), (73), (77), (90), (101), (72), (66), (109)	—
Triglycerides	(59), (85), (62), (69), (70), (71), (77), (81), (93), (75), (56)	(59), (73), (81), (90), (99), (100), (101), (105), (72), (66), (79), (109)	—
Total cholesterol	(59), (62), (69), (70), (71), (81), (81), (93), (99), (100), (101), (105), (75), (79), (56)	(73), (77), (81), (90), (72), (66, 109)	—
Lipoprotein A	(106), (70)	—	—
ApoA	—	(62), (79)	—
ApoB	(62), (69), (79)	—	—
*Endothelial function*			
Brachial artery diameter	(65)	—	—
Flow-mediated dilatation	(116), (86), (95), (108)	(85), (62), (65), (56)	—
*Blood pressure*			
Systolic blood pressure (trials)	(55), (73), (78), (112), (66), (77)	(63), (69), (71), (80), (89), (72), (79), (109), (62), (85)	—
Diastolic blood pressure (trials)	(63), (78), (112), (77)	(85), (62), (69), (71), (73), (80), (89), (55), (72), (79), (109), (66)	—
Hypertension (observational)	(107), (41), (118)	—	—
*Body composition and weight*			
Body composition	—	(74)	—
Body weight	(120), (121), (122), (123), (68, 87), (124)	(125), (124), (68), (115), (64), (74)	—
BMI	(123), (115), (124)	(121), (124), (68), (64), (74)	—
Energy intake	(125)	—	—
Fat mass	(68), (121)	(124), (68)	—
Overweight/obesity risk	(120), (121)	—	—
Waist circumference	(120), (124)	(60), (121), (68), (115), (64), (74)	—
*Hunger and fullness*			
Fullness	—	(125)	—
Hunger	(125)	—	—
Leptin	(84)	—	—
*Glucose and insulin*			
Fasting blood glucose	(60), (97), (98), (60), (123), (88)	(74)	—
Glycemic control	(88), (114), (123)	—	—
Insulin sensitivity	(98)	—	—
Fasting plasma insulin	(98)	(97)	—
Adiponectin	—	(84), (94)	—
HOMA-IR	(123), (88)	(74)	—
HbA1c		(97), (98), (88)	—
Glycemic indices	(92)	(74), (92)	—
*Inflammation*			
C-reactive protein	(58), (102)	(84), (95), (102), (103), (113)	—
TNF-α	(58), (102)	(84), (95)	—
IL-6, IL-10	(58)	(84), (95), (102)	—
Vascular, intercellular, andendothelial-leukocyte celladhesion proteins 1 (VCAM-1,ICAM-1, E-selectin)	(58)	(95)	—
Antioxidant defense system	(67), (129)	—	—
*Gut microbiota*			
Fecal microbiota	—	(24), (25)	—
*Cognitive function*			
Cognitive performance	(54)	(110)	—

1 Studies are categorized based on biomarker change [favorable/reduced disease risk, neutral (no significant change), or unfavorable/increased risk], and listed by reference number. HbA1c, glycated hemoglobin; ICAM-1, intercellular adhesion molecule 1; VCAM-1, vascular cell adhesion molecule 1.

### Diabetes, obesity, and metabolic disease

Meta-analyses reported mixed associations between high compared with low consumption of nuts and diabetes, obesity, and metabolic disease (15, 34, 37, 42, 43, 47, 51) (**Supplemental Figures 8** and **9**). For per serving data, a daily intake of 28 g nuts was associated with an RR of 0.89 (95% CI: 0.71–1.12; *I*^2^: 77%; AMSTAR-2: high) for diabetes mellitus type 2, and 0.61 (95% CI: 0.43–0.87; *I*^2^: 0%; AMSTAR-2: high) for diabetes-related mortality. For obesity there was a nonsignificant association (RR: 0.81; 95% CI: 0.62–1.07; *I*^2^: 74%; AMSTAR-2: moderate), whereas when assessing obesity/overweight, a significant association (RR: 0.89; 95% CI: 0.83–0.94; *I*^2^: 0%; AMSTAR-2: moderate) was observed. There were no significant nonlinear dose–response associations between consumption of nuts and diabetes (**Supplemental Figure 10**). There were 27,016 cases in the analysis of nut consumption and type 2 diabetes incidence.

Neither tree nuts nor peanuts separately were significantly associated with diabetes mortality. Most meta-analyses were adjusted for BMI, and these results might have been overadjusted. When not adjusting for BMI, an association between diabetes and nut consumption was seen, with an RR of 0.80 (95% CI: 0.69–0.94; *I*^2^: 51%, 2 large cohorts; AMSTAR-2: high), which could indicate that weight reduction might be a potential effect mediator for a potential effect on diabetes incidence (119).

#### Mechanisms related to diabetes, obesity, and metabolic disease

Trials and cohort studies have shown that diets enriched with nuts do not increase body weight, BMI, or waist circumference (60, 64, 68, 74, 87, 115, 120–125), with a tendency to a slight reduction in all of these. Overall, nuts and seeds showed a favorable trend in improving fasting blood glucose concentrations, glycemic control, and insulin sensitivity (60, 74, 88, 92, 97, 98, 114, 123). Furthermore, nuts have been found to contribute positively to satiety and reducing hunger (125), which might be one of the reasons studies have not found nuts to be linked with obesity (115, 126).

### Cancer

Meta-analyses indicated substantial inverse associations between high compared with low consumption of nuts and cancer-related mortality (16, 32–35) (**Supplemental Figures 11** and **12**). For per serving data, a daily intake of 28 g/d nuts was associated with an RR of 0.89 (95% CI: 0.83–0.94; *I*^2^: 23%; AMSTAR-2: moderate) for cancer mortality. For a meta-analysis on cancer mortality, 1 serving of 28 g/d was associated with the lowest risk of cancer mortality (Figure 4; **Supplemental Figure 13**). There were 49,161 cancer mortality cases in the overall analysis for all nuts.

There was a tendency toward stronger associations between tree nuts and cancer mortality than for peanuts. There is more uncertainty regarding the association between nut consumption and specific cancers. However, inverse associations were reported between nut consumption and endometrial, colon, pancreatic, gastric, and lung cancers, with less clear associations for rectal, esophageal, liver, endometrial, prostate, and breast cancer (35, 127, 128).

#### Mechanisms related to cancer

Trials assessing inflammatory outcomes from nut consumption have generally found a slightly favorable or neutral change in inflammatory markers such as C-reactive protein, ILs, TNF-α, cell adhesion molecules, and antioxidant defense system (58, 67, 84, 95, 102, 103, 113, 129–132). Further, insulin sensitivity, glycemic control, and obesity (see above) might also be relevant for cancers.

### All-cause mortality and other cause-specific mortality

Meta-analyses have shown substantial inverse associations between nut intake and all-cause mortality (**Supplemental Figures 14** and **15**) (13, 14, 16, 33, 40, 43, 45, 46). A daily intake of 28 g/d nuts was associated with an RR of 0.78 (95% CI: 0.72–0.84; *I*^2^: 66%; AMSTAR-2: high), with the dose–response curves plateauing from ∼20 g/d (**Supplemental Figure 16**). There were 819,448 participants and 85,870 deaths in the mortality analyses. There were no clear differences in mortality outcomes between tree nuts and peanuts.

Relating to other cause-specific mortality, there were also observed inverse associations between nut consumption and mortality from respiratory disease (RR: 0.48; 95% CI: 0.26–0.89; *I*^2^: 61%; AMSTAR-2: high), and infectious disease (RR: 0.25; 95% CI: 0.07–0.87; *I*^2^: 54%; AMSTAR-2: high) (16) (**Supplemental Figures 17** and **18**). A nonsignificant association was observed for neurodegenerative disease mortality (RR: 0.65; 95% CI: 0.40–1.07; *P*  = 0.086; *I*^2^: 6%; AMSTAR-2: high). No significant association was seen for kidney-related disease mortality. For these 4 outcomes, 2551/397/367/2056 deaths were included in each analysis.

### Allergy and adverse reactions to nuts and seeds

Nine meta-analyses and systematic review articles provided data on allergies and adverse reactions. Using the gold standard diagnostic methods of peanut allergy, the prevalence ranged between 0% to 2.8%, with heterogeneity between age groups and settings (9, 133–135) (**Supplemental Table 4**). Food challenge tests indicated the following age-specific prevalence of allergies to tree nuts: 0–6 y: 0.03–0.2%; 6–18 y: 0.2–2.3%; and adults: 0.4–1.4% (136). Challenge-proven data are sparse for non-European countries. Anaphylactic reactions were rare, but among these peanut seems to be the leading food allergen (22), and can be life-threatening if not handled promptly and correctly. Among individuals with peanut allergy, 1 to 6 anaphylaxis events are estimated per 2500 patients exposed to low-dose nut protein (137). Consumption of cashew nuts is also a relatively common cause of anaphylactic reactions, often with cross-reactions to pistachio nuts (138). The prevalence of allergy to sesame seeds was estimated as 0.1–0.2% (139).

## Discussion

An intake of 28 g/d nuts compared with not eating nuts was associated with a 21% RR reduction for cardiovascular disease (including coronary heart disease incidence and mortality, atrial fibrillation, and stroke mortality), 11% risk reduction for cancer deaths, and 22% reduction for all-cause mortality. Nut consumption was also associated with a reduced risk of mortality from respiratory diseases, infectious diseases, and diabetes; however, associations between nut consumption and diabetes incidence were mixed. Generally, these associations seem to be relatively similar for different nuts, including different tree nuts and peanuts. Meta-analyses of trials on biomarkers for disease (intermediate factors) generally mirrored meta-analyses from observational studies on cardiovascular disease, cancer mortality, and diabetes. Dose–response relations suggest optimal intake levels of 15–40 g/d with generally limited benefits in increasing intake beyond 28 g/d.

We observed mixed associations between consumption of nuts and diabetes incidence or diabetes mortality. It is possible that a potential inverse association between consumption of nuts and diabetes incidence could largely be mediated by body weight, and that meta-analyses adjusting for BMI might have overadjusted analyses masking associations between consumption of nuts and diabetes incidence (119). This assumption is supported by studies that generally showed weaker associations between nuts and diabetes incidence when adjusting for BMI (119). Consumption of a handful of nuts per day is unlikely to contribute to overweight and obesity based on the current evidence (126). Around half of the meta-analyses conducted indicated slightly favorable effects of nuts on body weight and fat mass.

Allergies for nuts are reported in ∼1–2%, with substantial heterogeneity between populations (134). Allergies to seeds are relatively uncommon (139). Severe allergic reactions, and particularly anaphylactic reactions, can be life-threatening if not handled promptly and correctly (9, 138). However, many reactions are also milder cross-reactions (140). Roasting generally reduces the allergenicity of some nut allergies (e.g., hazelnut and almonds) (140). Because avoidance of known allergens is the cornerstone for people with allergies, labeling of food to ensure transparency is essential (22). Legislation for allergen disclosure generally reflects allergens commonly responsible for food anaphylaxis (133). Some nuts, such as Brazil nuts, are more prone to contain potentially harmful fungal toxins (such as aflatoxin) when stored after inadequate drying (23). The presence of such toxins can generally be limited by regulations in the processing and distribution of nuts (23).

The current evidence strongly supports nut consumption as part of a healthy but also sustainable diet, in terms of greenhouse gas emissions, land and energy use, and potential for acidification and eutrophication (141–146). Furthermore, an increased intake of nuts to ≥20 g/d could have averted 4.4 million deaths in North and South America, Europe, Southeast Asia, and the Western Pacific (16). This is estimated from probable reductions in premature deaths related to cardiovascular disease and cancers and possible reductions in mortality from respiratory disease and diabetes. Some systematic reviews have further suggested that nut consumption is positively associated with cognitive function tests (54, 147), and nuts might have a role both in child development and in slowing some age-related cognitive decline. For children, less evidence is available relating to the effect of nut consumption on disease patterns, but the studies generally show some similarities in trends to what is presented for adults (148). Intake amounts can be adapted to the individual's age, and the youngest children generally need less energy. Recommending an intake of a handful of nuts per day will to some degree take this into consideration because hand size will also grow relatively parallel with body size.

This study has several strengths and some limitations. This is the most comprehensive umbrella review conducted on nut and seed consumption and its associations with disease and mortality outcomes. For many of the included outcomes, no umbrella reviews are presently available, and for the remainder, many studies were published subsequent to the relevant umbrella reviews. We have included both disease and mortality outcomes, and biomarkers for disease as intermediate outcomes with mechanistic studies to better identify causal effects. We have strived to adhere to the PRISMA criteria (31). Still, some data might have been missed due to inadequate indexing in MEDLINE and Embase, or titles and abstracts not indicating the articles to be relevant. The former is more common for older studies, but these are probably few. Some trials have included nuts as a component of a complex intervention (83, 149). For several of the interventions with several components that can contribute to the outcomes of interest, the duration of these trials might also have been too short to achieve relevant effects on many of these chronic diseases (83, 150). Relating to cancer, one might argue against assessing all cancers combined because cancers are heterogeneous. On the other hand, cancers generally share a range of mechanisms, and assessing all cancers separately increases the risk of random variability errors. In meta-analyses on cancer incidence, there might have been studies including data on assessing cancer-related mortality (mixing different outcomes). Thus, the validity of the cancer incidence is uncertain, and some studies have questioned whether nut intake is associated with cancer incidence (150, 151). However, there is more agreement on the inverse associations between nut intake and cancer mortality. There are also many studies on diet patterns that include nuts but do not assess the effect of nuts and other food groups individually. Our assessment omitted these because it is difficult to ascribe effects to separate food groups. Finally, inadequately described study methods, such as lacking specification, might have been the cause for the rejection of otherwise relevant studies. Double controlling has contributed to preventing mistakes, and when there is room for different interpretations, these have been discussed among the authors.

## Conclusion

Intake of nuts is inversely associated with the risk of cardiovascular diseases. This is mirrored with experimental studies on biomarkers for cardiovascular disease, with the overall quality of evidence considered moderate. Compared with not eating nuts, a handful of nuts per day is associated with a risk reduction of cardiovascular disease and mortality by a fifth, and cancer deaths by a tenth. Nut consumption is also associated with a substantial reduction in mortality risk from respiratory diseases, infectious diseases, and diabetes; however, associations between nut consumption and diabetes incidence are mixed and might be explained by adiposity differences. Meta-analyses of trials on intermediate factors of other chronic diseases also generally mirror meta-analyses from observational studies on cardiovascular, cancer, metabolic, and infectious diseases.

Hence, the current evidence supports dietary recommendations to consume a handful of nuts and seeds per day for people without allergies to these foods. Different types of nuts and seeds seem to have broadly similar benefits.

## Supplementary Material

nmac077_Supplemental_File

## Notes

The authors were funded by their respective institutions. The study had no additional funding. The funders had no role in study design, data collection and analysis, decision to publish, or preparation of the manuscript.

Author disclosures: The authors report no conflicts of interest.

Supplemental Figures 1–18, Supplemental Tables 1–4, and Supplemental Material are available from the “Supplementary data” link in the online posting of the article and from the same link in the online table of contents at https://academic.oup.com/advances/.
