# Supplementary material for: Consumption of Nuts and Seeds and Health Outcomes Including Cardiovascular Disease, Diabetes and Metabolic Disease, Cancer, and Mortality: An Umbrella Review
Source: Adv Nutr. 2022 Aug 30;13(6):2136–48. doi: 10.1093/advances/nmac077 (PMC9776667; doi:10.1093/advances/nmac077)
Supplement: nmac077_Supplemental_File [file nmac077_supplemental_file.pdf]

Consumption of nuts and seeds and health outcomes including cardiovascular, diabetes and metabolic disease, cancer, and mortality: an umbrella review

Rajiv Balakrishna, Tonje Bjørnerud, Mitra Bemanian, Dagfinn Aune, Lars T. Fadnes*

Online Supplementary Material

* Corresponding author: Lars Fadnes, email: Lars.Fadnes@uib.no, +4755970173, Address: Post box 7804, 5020 Bergen, Norway.

Supplementary file including the following data:
Supplementary table 1: List of outcome measures from meta-analyses on nut consumption and associations with various morbidities and mortalities (e.g. couting per serving relationship and high/low separately, similarly also for all nuts vs. subtypes of nuts).

Supplementary table 2: Assessing the methodological quality of systematic reviews with meta-analysis outcomes on nut consumption and associations to diseases and mortality with overall rating and assessment on sixteen criteria listed below the table.

Supplementary table 3: Summary of systematic reviews with meta-analysis on nut consumption and associations to diseases and mortality with information of search year, nut type, outcome, number of studies, unit (high versus low [HL] or per serving/dose expressed in gram per day), relative risk ratios, number of participants and cases, heterogeneity, and overall AMSTAR-2 evaluation.

Supplementary table 4: Summary of data on adverse reactions including allergy and anaphylaxis on nut and seed consumption.

Supplementary figure 1: Summary of associations from the most recent meta-analyses between high compared to low consumption of nuts and risk of various morbidities and mortalities. Reference number is listed in brackets and search year is listed within the parentheses.

Supplementary figure 2: Summary of per serving associations from the most recent meta-analyses between consumption of 28g nuts and risk of various morbidities and mortalities. Reference number is listed in brackets and search year is listed within the parentheses.

Supplementary figure 3: Summary of associations from meta-analyses between high compared to low consumption of nuts and risk of various cardiovascular morbidities and mortalities. Reference number is listed in brackets and search year is listed within the parentheses. Number of studies, participants (n) and cases, as well as heterogeneity (I^2) is also listed in the right column.

Supplementary figure 4: Summary of associations from meta-analyses between per serving relationship of 28g consumption of nuts and risk of various cardiovascular morbidities and mortalities. Reference number is listed in brackets and search year is listed within the parentheses. Number of studies, participants (n) and cases, as well as heterogeneity (I^2) is also listed in the right column.

Supplementary figure 5: Summary of associations from meta-analyses between high compared to low consumption of nuts and risk of mortality. Reference number is listed in brackets and search year is listed within the parentheses. Number of studies, participants (n) and cases, as well as heterogeneity (I^2) is also listed in the right column.

Supplementary figure 6: Summary of associations from meta-analyses between per serving relationship of 28g consumption of nuts and risk of mortality. Reference number is listed in brackets and search year is listed within the parentheses. Number of studies, participants (n) and cases, as well as heterogeneity (I^2) is also listed in the right column.

Supplementary figure 7: Summary of associations from meta-analyses between high compared to low consumption of nuts and risk of cancer and cancer-related mortality. Reference number is listed in brackets and search year is listed within the parentheses. Number of studies, participants (n) and cases, as well as heterogeneity (I^2) is also listed in the right column.

Supplementary figure 8: Summary of associations from meta-analyses between per serving relationship of 28g consumption of nuts and risk of cancer and cancer-related mortality. Reference number is listed in brackets and search year is listed within the parentheses. Number of studies, participants (n) and cases, as well as heterogeneity (I^2) is also listed in the right column.

Supplementary figure 9: Summary of associations from meta-analyses between high compared to low consumption of nuts and risk of various diabetes-related morbidities and mortalities. Reference number is listed in brackets and search year is listed within the parentheses. Number of studies, participants (n) and cases, as well as heterogeneity (I^2) is also listed in the right column.

Supplementary figure 10: Summary of associations from meta-analyses between per serving relationship of 28g consumption of nuts and risk of various diabetes-related morbidities and mortalities. Reference number is listed in brackets and search year is listed within the parentheses. Number of studies, participants (n) and cases, as well as heterogeneity (I^2) is also listed in the right column.

Supplementary figure 11: Summary of associations from meta-analyses between high compared to low consumption of nuts and risk of various other morbidities and mortalities. Reference number is listed in brackets and search year is listed within the parentheses. Number of studies, participants (n) and cases, as well as heterogeneity (I^2) is also listed in the right column.

Supplementary figure 12: Summary of associations from meta-analyses between per serving relationship of 28g consumption of nuts and risk of various other morbidities and mortalities. Reference number is listed in brackets and search year is listed within the parentheses. Number of studies, participants (n) and cases, as well as heterogeneity (I^2) is also listed in the right column.

Supplementary figure 13: Summary of dose-response relationships from meta-analyses on associations between nut consumption of nuts and risk of cardiovascular diseases. The relationships are grouped by meta-analysis (labelled by first author).

Supplementary figure 14: Summary of dose-response relationships from meta-analyses on associations between nut consumption of nuts and risk of coronary heart/artery disease. The relationships are grouped by meta-analysis (labelled by first author).

Supplementary figure 15: Summary of dose-response relationships from meta-analyses on associations between nut consumption of nuts and risk of stroke diseases. The relationships are grouped by meta-analysis (labelled by first author).

Supplementary figure 16: Summary of dose-response relationships from meta-analyses on associations between nut consumption of nuts and risk of diabetes. The relationships are grouped by meta-analysis (labelled by first author).

Supplementary figure 17: Summary of dose-response relationships from meta-analyses on associations between nut consumption of nuts and risk of cancer. The relationships are grouped by meta-analysis (labelled by first author).

Supplementary figure 18: Summary of dose-response relationships from meta-analyses on associations between nut consumption of nuts and risk of mortality. The relationships are grouped by meta-analysis (labelled by first author).

Supplementary text 1: Search documentation for literature search

Supplementary table 1: List of outcome measures from meta-analyses on nut consumption and associations with various morbidities and mortalities (e.g. couting dose-relationship and high/low separately, similarly also for all nuts vs. subtypes of nuts).

Atrial fibrillation	1
Coronary heart disease	20
Coronary heart disease mortality	11
Cardiovascular disease	13
Cardiovascular disease mortality	13
Cancer mortality	21
Diabetes mellitus	11
Diabetes mortality	6
Heart failure	3
Hemorrhagic stroke incidence	4
Infectious disease mortality	6
Ischemic stroke incidence	4
Kidney disease mortality	6
Metabolic syndrome	4
Mortality	19
Neurodegenerative disease mortality	6
Obesity	1
Overweight or obesity-	1
Respiratory disease mortality	7
Stroke	20
Stroke mortality	13

Supplementary table 2: Assessing the methodological quality of systematic reviews with meta-analysis outcomes on nut consumption and associations to diseases and mortality with overall rating and assessment on sixteen criteria listed below the table.

Reference number	First author	1	2	3	4	5	6	7	8	9	10	11	12	13	14	15	16	Overall Rating
37	Afshin	Yes	No	Yes	Yes	No	Yes	Partly	Yes	Yes	Yes	Yes	Yes	Yes	Yes	Yes	Yes	Moderate
16	Aune	Yes	Partly	Yes	Yes	Yes	Yes	Yes	Yes	Yes	Yes	Yes	Yes	Yes	Yes	Yes	Yes	High
15	Becerra-Tomás	Yes	Yes	Yes	Yes	Yes	Yes	Partly	Yes	Yes	Yes	Yes	Yes	Yes	Yes	Yes	Yes	High
38	Becerra-Tomás	Yes	Yes	Yes	Yes	Yes	Yes	Partly	Yes	Yes	Yes	Yes	Yes	Yes	Yes	Yes	Yes	High
39	Bechthold	Yes	Yes	Yes	Yes	Yes	Yes	Yes	Yes	Yes	Yes	Yes	Yes	Yes	Yes	Yes	Yes	High
40	Brandt	Yes	No	Yes	Partly	No	No	No	No	Yes	Yes	Yes	No	Yes	Yes	Yes	Yes	Low
13	Chen	Yes	No	Yes	Yes	Yes	Yes	Partly	Yes	Yes	Yes	Yes	Yes	Yes	Yes	Yes	Yes	Moderate
14	Grosso	Yes	No	Yes	Yes	Yes	Yes	Partly	Yes	Yes	Yes	Yes	Yes	Yes	Yes	Yes	Yes	Moderate
41	Guo	Yes	No	Yes	Yes	Yes	Yes	Partly	Yes	Yes	Yes	Yes	Yes	Yes	Yes	Yes	Yes	Moderate
42	Li	Yes	Partly	Yes	Yes	Yes	Yes	Yes	Yes	Yes	Yes	Yes	Yes	Yes	Yes	Yes	Yes	High
43	Luo	Yes	Partly	Yes	Yes	Yes	Yes	Partly	Yes	Yes	Yes	Yes	Yes	Yes	Yes	Yes	Yes	High
44	Ma	Yes	No	Yes	Yes	Yes	Yes	Yes	Yes	Yes	No	Yes	No	Yes	Yes	Yes	Yes	Low
45	Mayhew	Yes	No	Yes	Yes	Yes	Yes	Partly	Yes	Yes	Yes	Yes	Yes	Yes	Yes	Yes	Yes	Moderate
33	Naghshi	Yes	Partly	Yes	Yes	Yes	Yes	Yes	Yes	Yes	Yes	Yes	Yes	Yes	Yes	Yes	Yes	High
46	Schwingshackl	Yes	Yes	Yes	Yes	Yes	Yes	Partly	Yes	Yes	Yes	Yes	Yes	Yes	Yes	Yes	Yes	High
47	Schwingshackl	Yes	Yes	Yes	Yes	Yes	Yes	Partly	Yes	Yes	Yes	Yes	Yes	Yes	Yes	Yes	Yes	High
48	Shao	Yes	Partly	Yes	Yes	Yes	Yes	Yes	Yes	Yes	No	Yes	Yes	Yes	Yes	Yes	Yes	Moderate
49	Shi	Yes	No	Yes	Yes	Yes	Yes	Partly	Yes	Yes	Yes	Yes	Yes	Yes	Yes	Yes	Yes	Moderate
50	Weng	Yes	No	Yes	Yes	Yes	Yes	Partly	Yes	Yes	Yes	Yes	Yes	Yes	Yes	Yes	Yes	Moderate
34	Wu	Yes	Yes	Yes	Yes	Yes	Yes	Partly	Yes	No	Yes	Yes	No	No	Yes	Yes	Yes	Low
51	Zhang	Yes	Partly	Yes	Yes	Yes	Yes	Yes	Yes	No	Yes	Yes	Yes	Yes	Yes	Yes	Yes	Moderate
35	Zhang	Yes	Partly	Yes	Yes	Yes	Yes	Partly	Yes	Yes	Yes	Yes	Yes	Yes	Yes	Yes	Yes	High
52	Zhang	Yes	No	Yes	Yes	Yes	Yes	Partly	Yes	Yes	Yes	Yes	Yes	Yes	Yes	Yes	Yes	Moderate

1. PICO (Did the research questions and inclusion criteria for the review include the components of PICO? - Population, Intervention, Comparator group, Outcome)

2. Protocol (Did the report of the review contain an explicit statement that the review methods were established prior to the conduct of the review and did the report justify any significant deviations from the protocol?)
3. Design argued (Did the review authors explain their selection of the study designs for inclusion in the review?)
4. Search and database selection (Did the review authors use a comprehensive literature search strategy?)
5. Duplicate study selection (Did the review authors perform study selection in duplicate?)
6. Duplicate extraction (Did the review authors perform data extraction in duplicate?)
7. Justify exclusion (Did the review authors provide a list of excluded studies and justify the exclusions?)
8. PICO details (Did the review authors describe the included studies in adequate detail?)
9. Bias/confounding (Did the review authors use a satisfactory technique for assessing the risk of bias (RoB) in individual studies that were included in the review?)
10. Funding (Did the review authors report on the sources of funding for the studies included in the review?)
11. If meta-analysis, statistical methods (If meta-analysis was performed did the review authors use appropriate methods for statistical combination of results?)
12. Bias - sensitivity (If meta-analysis was performed, did the review authors assess the potential impact of RoB in individual studies on the results of the meta-analysis or other evidence synthesis?)
13. Bias discussed/accounted (Did the review authors account for RoB in individual studies when interpreting/ discussing the results of the review?)
14. Heterogeneity discussed (Did the review authors provide a satisfactory explanation for, and discussion of, any heterogeneity observed in the results of the review?)
15. Publication bias (If they performed quantitative synthesis did the review authors carry out an adequate investigation of publication bias (small study bias) and discuss its likely impact on the results of the review?)
16. Conflicts of interest (Did the review authors report any potential sources of conflict of interest, including any funding they received for conducting the review?)

Supplementary table 3: Summary of systematic reviews with meta-analysis on nut consumption and associations to diseases and mortality with information of search year, nut type, outcome, number of studies, unit (high versus low [HL] or dose response expressed in gram per day), relative risk ratios, number of participants and cases, heterogeneity, and overall AMSTAR-2 evaluation.

First author	Search year	Ref. no.	Nut types	Outcome	Unit	Relative risk ratio (CI)	Participants / cases (studies)	I2	AMSTAR-2
Afshin	2013	37	All nuts	CHD	15g	0.78 (0.67-0.92)	141390 / 2101 (4)	0	Moderate
Afshin	2013	37	All nuts	CHD mortality	15g	0.76 (0.69-0.84)	206114 / 6749 (4)	28	Moderate
Afshin	2013	37	All nuts	Stroke	15g	0.89 (0.74-1.05)	155685 / 5544 (4)	73	Moderate
Afshin	2013	37	All nuts	Diabetes mellitus	15g	0.87 (0.81-0.94)	230216 / 13308 (6)	22	Moderate
Aune	2016	16	All nuts	CVD	HL	0.81 (0.74-0.89)	376228 / 18655 (11)	52	High
Aune	2016	16	All nuts	CHD	HL	0.76 (0.69-0.84)	315397 / 12331 (11)	48	High
Aune	2016	16	All nuts	Stroke	HL	0.89 (0.82-0.97)	396768 / 9272 (10)	0	High
Aune	2016	16	All nuts	Mortality	HL	0.81 (0.77-0.85)	819448 / 85870 (15)	41	High
Aune	2016	16	All nuts	Cancer mortality	HL	0.82 (0.74-0.89)	254240 / 17603 (8)	28	High
Aune	2016	16	All nuts	Diabetes mortality	HL	0.68 (0.52-0.9)	202751 / 800 (4)	0	High
Aune	2016	16	All nuts	Respiratory disease mortality	HL	0.76 (0.61-0.94)	130987 / 2551 (3)	54	High
Aune	2016	16	All nuts	Infectious disease mortality	HL	0.79 (0.56-1.11)	118962 / 397 (2)	0	High
Aune	2016	16	All nuts	Kidney disease mortality	HL	0.69 (0.38-1.25)	118962 / 367 (2)	69	High
Aune	2016	16	All nuts	Neurodegenerative dis. mortality	HL	0.93 (0.72-1.21)	130987 / 2056 (3)	6	High
Aune	2016	16	Tree nuts	CVD	HL	0.81 (0.74-0.89)	130987 / 9456 (3)	0	High
Aune	2016	16	Tree nuts	CHD	HL	0.79 (0.68-0.92)	130987 / 6394 (3)	28	High
Aune	2016	16	Tree nuts	Stroke	HL	0.89 (0.82-0.97)	130987 / 2130 (3)	0	High
Aune	2016	16	Tree nuts	Mortality	HL	0.8 (0.74-0.86)	202751 / 42508 (4)	58	High
Aune	2016	16	Tree nuts	Cancer mortality	HL	0.82 (0.76-0.9)	130987 / 14210 (3)	0	High
Aune	2016	16	Tree nuts	Diabetes mortality	HL	1.19 (0.74-1.89)	130987 / 462 (3)	0	High
Aune	2016	16	Tree nuts	Respiratory disease mortality	HL	0.89 (0.74-1.07)	130987 / 2551 (3)	0	High
Aune	2016	16	Tree nuts	Infectious disease mortality	HL	0.73 (0.47-1.13)	118962 / 397 (2)	0	High
Aune	2016	16	Tree nuts	Kidney disease mortality	HL	0.65 (0.4-1.03)	118962 / 367 (2)	0	High
Aune	2016	16	Tree nuts	Neurodegenerative dis. mortality	HL	0.94 (0.75-1.18)	130987 / 2056 (3)	14	High
Aune	2016	16	Peanuts	CVD	HL	0.81 (0.75-0.87)	265252 / 12043 (5)	15	High
Aune	2016	16	Peanuts	CHD	HL	0.76 (0.69-0.82)	265252 / 7025 (5)	0	High
Aune	2016	16	Peanuts	Stroke	HL	0.83 (0.69-1)	265252 / 3315 (5)	46	High
Aune	2016	16	Peanuts	Mortality	HL	0.85 (0.82-0.89)	265252 / 44396 (5)	18	High
Aune	2016	16	Peanuts	Cancer mortality	HL	0.93 (0.87-0.99)	265252 / 17742 (5)	19	High
Aune	2016	16	Peanuts	Diabetes mortality	HL	0.84 (0.6-1.19)	202751 / 800 (4)	43	High
Aune	2016	16	Peanuts	Respiratory disease mortality	HL	0.77 (0.63-0.93)	130987 / 2551 (3)	39	High

Aune	2016	16	Peanuts	Infectious disease mortality	HL	1.01 (0.83-1.23)	118962 / 397 (2)	13	High
Aune	2016	16	Peanuts	Kidney disease mortality	HL	0.52 (0.27-0.97)	118962 / 367 (2)	0	High
Aune	2016	16	Peanuts	Neurodegenerative dis. mortality	HL	0.94 (0.72-1.23)	130987 / 2056 (3)	46	High
Aune	2016	16	All nuts	CVD	28g	0.79 (0.7-0.88)	376228 / 18655 (12)	60	High
Aune	2016	16	All nuts	CHD	28g	0.71 (0.63-0.8)	315397 / 12331 (11)	47	High
Aune	2016	16	All nuts	Stroke	28g	0.93 (0.83-1.05)	396768 / 9272 (11)	14	High
Aune	2016	16	All nuts	Mortality	28g	0.78 (0.72-0.84)	819448 / 85870 (16)	66	High
Aune	2016	16	All nuts	Cancer mortality	28g	0.85 (0.76-0.94)	254240 / 17603 (8)	42	High
Aune	2016	16	All nuts	Diabetes mortality	28g	0.61 (0.43-0.88)	202751 / 800 (4)	0	High
Aune	2016	16	All nuts	Respiratory disease mortality	28g	0.48 (0.26-0.89)	130987 / 2551 (3)	61	High
Aune	2016	16	All nuts	Infectious disease mortality	28g	0.25 (0.07-0.85)	118962 / 397 (2)	54	High
Aune	2016	16	All nuts	Kidney disease mortality	28g	0.27 (0.04-1.91)	118962 / 367 (2)	61	High
Aune	2016	16	All nuts	Neurodegenerative dis. mortality	28g	0.65 (0.4-1.08)	130987 / 2056 (3)	6	High
Aune	2016	16	Tree nuts	CVD	28g	0.75 (0.67-0.84)	130987 / 9456 (3)	0	High
Aune	2016	16	Tree nuts	CHD	28g	0.73 (0.63-0.85)	130987 / 6394 (3)	0	High
Aune	2016	16	Tree nuts	Stroke	28g	0.89 (0.69-1.14)	130987 / 2130 (3)	0	High
Aune	2016	16	Tree nuts	Mortality	28g	0.82 (0.75-0.9)	202751 / 42508 (4)	70	High
Aune	2016	16	Tree nuts	Cancer mortality	28g	0.8 (0.72-0.89)	130987 / 14210 (3)	0	High
Aune	2016	16	Tree nuts	Diabetes mortality	28g	1.23 (0.68-2.25)	130987 / 462 (3)	0	High
Aune	2016	16	Tree nuts	Respiratory disease mortality	28g	0.79 (0.62-1.01)	130987 / 2551 (3)	0	High
Aune	2016	16	Tree nuts	Infectious disease mortality	28g	0.64 (0.36-1.13)	118962 / 397 (2)	0	High
Aune	2016	16	Tree nuts	Kidney disease mortality	28g	0.66 (0.36-1.22)	118962 / 367 (2)	0	High
Aune	2016	16	Tree nuts	Neurodegenerative dis. mortality	28g	0.81 (0.58-1.12)	130987 / 2056 (3)	26	High
Aune	2016	16	Peanuts	CVD	28g	0.64 (0.5-0.81)	265252 / 12043 (5)	77	High
Aune	2016	16	Peanuts	CHD	28g	0.69 (0.57-0.84)	265252 / 7025 (5)	45	High
Aune	2016	16	Peanuts	Stroke	28g	0.63 (0.41-0.95)	265252 / 3315 (5)	78	High
Aune	2016	16	Peanuts	Mortality	28g	0.77 (0.69-0.86)	265252 / 44396 (5)	64	High
Aune	2016	16	Peanuts	Cancer mortality	28g	0.92 (0.82-1.03)	265252 / 17742 (5)	30	High
Aune	2016	16	Peanuts	Diabetes mortality	28g	0.73 (0.45-1.2)	202751 / 800 (4)	15	High
Aune	2016	16	Peanuts	Respiratory disease mortality	28g	0.69 (0.53-0.91)	130987 / 2551 (3)	50	High
Aune	2016	16	Peanuts	Infectious disease mortality	28g	1.03 (0.81-1.31)	118962 / 397 (2)	3	High
Aune	2016	16	Peanuts	Kidney disease mortality	28g	0.42 (0.24-0.73)	118962 / 367 (2)	0	High
Aune	2016	16	Peanuts	Neurodegenerative dis. mortality	28g	0.92 (0.65-1.31)	130987 / 2056 (3)	49	High
Becerra-Tomás	2019	38	All nuts	CVD	HL	0.85 (0.8-0.91)	210836 / 14136 (3)	0	High
Becerra-Tomás	2019	38	All nuts	CVD mortality	HL	0.77 (0.72-0.82)	413727 / 14475 (14)	3	High
Becerra-Tomás	2019	38	All nuts	CHD	HL	0.82 (0.69-0.96)	275812 / 12654 (7)	74	High
Becerra-Tomás	2019	38	All nuts	CHD mortality	HL	0.76 (0.67-0.86)	396041 / 7877 (12)	46	High

Becerra-Tomás	2019	38	All nuts	Stroke	HL	1 (0.92-1.09)	302888 / 12646 (7)	0	High
Becerra-Tomás	2019	38	All nuts	Stroke mortality	HL	0.83 (0.75-0.93)	351618 / 2332 (11)	0	High
Becerra-Tomás	2019	38	All nuts	Hemorrhagic stroke incidence	HL	1.02 (0.77-1.34)	188750 / 3088 (5)	15	High
Becerra-Tomás	2019	38	All nuts	Ischemic stroke incidence	HL	0.99 (0.89-1.1)	302423 / 8401 (7)	0	High
Becerra-Tomás	2019	38	All nuts	Atrial fibrillation	HL	0.85 (0.73-0.99)	53965 / 10867 (2)	0	High
Becerra-Tomás	2019	38	All nuts	Heart failure	HL	1 (0.86-1.16)	53887 / 4253 (2)	0	High
Becerra-Tomás	2019	38	All nuts	CVD	28g	0.87 (0.81-0.93)	210836 / 14136 (3)	0	High
Becerra-Tomás	2019	38	All nuts	CVD mortality	28g	0.71 (0.61-0.84)	413727 / 14475 (14)	3	High
Becerra-Tomás	2019	38	All nuts	CHD	28g	0.75 (0.64-0.88)	275812 / 12654 (7)	74	High
Becerra-Tomás	2019	38	All nuts	CHD mortality	28g	0.67 (0.52-0.87)	396041 / 7877 (12)	46	High
Becerra-Tomás	2019	38	All nuts	Stroke	28g	1.06 (0.97-1.15)	302888 / 12646 (7)	0	High
Becerra-Tomás	2019	38	All nuts	Stroke mortality	28g	1.01 (0.88-1.18)	351618 / 2332 (11)	0	High
Becerra-Tomás	2019	38	All nuts	Hemorrhagic stroke incidence	28g	1.05 (0.77-1.43)	188750 / 3088 (5)	15	High
Becerra-Tomás	2019	38	All nuts	Ischemic stroke incidence	28g	1.06 (0.86-1.31)	302423 / 8401 (7)	0	High
Becerra-Tomás	2019	38	Tree nuts	CVD	HL	0.85 (0.79-0.91)	210836 / 14136 (3)	0	High
Becerra-Tomás	2019	38	Tree nuts	CHD	HL	0.77 (0.7-0.84)	210836 / 8390 (3)	61	High
Becerra-Tomás	2019	38	Tree nuts	Stroke	HL	1 (0.89-1.11)	210836 / 5910 (3)	0	High
Becerra-Tomás	2019	38	Tree nuts	Stroke mortality	HL	0.93 (0.77-1.13)	118962 / 1851 (3)	0	High
Becerra-Tomás	2019	38	Peanuts	CVD	HL	0.87 (0.81-0.93)	210836 / 14136 (3)	0	High
Becerra-Tomás	2019	38	Peanuts	CVD mortality	HL	0.77 (0.7-0.85)	134265 / 5572 (2)	0	High
Becerra-Tomás	2019	38	Peanuts	CHD	HL	0.85 (0.79-0.92)	210836 / 8390 (3)	0	High
Becerra-Tomás	2019	38	Peanuts	CHD mortality	HL	0.75 (0.64-0.88)	134265 / 2119 (2)	0	High
Becerra-Tomás	2019	38	Peanuts	Stroke	HL	0.9 (0.81-0.99)	210836 / 5910 (3)	13	High
Becerra-Tomás	2019	38	Peanuts	Stroke mortality	HL	0.83 (0.73-0.95)	253227 / 3036 (4)	57	High
Becerra-Tomás	2019	38	Walnuts	CVD	HL	0.81 (0.71-0.92)	144021 / 5255 (3)	3	High
Becerra-Tomás	2019	38	Walnuts	CHD	HL	0.79 (0.66-0.94)	144021 / 2685 (3)	4	High
Becerra-Tomás	2019	38	Walnuts	Stroke	HL	0.85 (0.71-1.02)	144021 / 5910 (3)	19	High
Becerra-Tomás	2021	15	All nuts	Diabetes mellitus	HL	1.04 (0.94-1.15)	194168 / (4)	60	High
Becerra-Tomás	2021	15	Tree nuts	Diabetes mellitus	HL	0.98 (0.87-1.11)	137956 / (2)	0	High
Becerra-Tomás	2021	15	Walnuts	Diabetes mellitus	HL	0.76 (0.62-0.94)	137956 / (1)	0	High
Becerra-Tomás	2021	15	Peanuts	Diabetes mellitus	HL	0.95 (0.87-1.04)	202147 / (3)	72	High
Bechthold	2017	39	All nuts	CHD	HL	0.8 (0.62-1.03)	/ 5480 (4)	79	High
Bechthold	2017	39	All nuts	Stroke	HL	0.94 (0.85-1.05)	/ 7490 (6)	18	High
Bechthold	2017	39	All nuts	Heart failure	HL	0.99 (0.86-1.15)	/ 3613 (3)	57	High
Bechthold	2017	39	All nuts	CHD	28g	0.67 (0.43-1.05)	/ 5480 (4)	85	High
Bechthold	2017	39	All nuts	Stroke	28g	0.99 (0.84-1.17)	/ 7490 (6)	45	High
Bechthold	2017	39	All nuts	Heart failure	28g	1.09 (0.97-1.22)	/ 3613 (2)	0	High

Brandt (van den)	2014	40	All nuts	Cancer mortality	HL	0.85 (0.77-0.93)	247030 / 14340 (4)	17	Low
Brandt (van den)	2014	40	All nuts	Respiratory disease mortality	HL	0.71 (0.58-0.86)	239814 / 2551 (3)	5	Low
Chen	2016	13	All nuts	CVD mortality	HL	0.75 (0.71-0.79)	524610 / 19574 (16)	0	Moderate
Chen	2016	13	All nuts	CHD mortality	HL	0.73 (0.67-0.8)	429833 / 10083 (13)	14	Moderate
Chen	2016	13	All nuts	Stroke mortality	HL	0.82 (0.73-0.91)	449293 / 4398 (12)	0	Moderate
Chen	2016	13	All nuts	Mortality	HL	0.81 (0.78-0.84)	498730 / 66568 (16)	22	Moderate
Chen	2016	13	All nuts	Cancer mortality	HL	0.87 (0.8-0.93)	451589 / 21353 (11)	26	Moderate
Chen	2016	13	Tree nuts	CVD mortality	HL	0.81 (0.74-0.89)	130987 / 9456 (3)	0	Moderate
Chen	2016	13	Tree nuts	CHD mortality	HL	0.79 (0.68-0.92)	130987 / 6394 (3)	28	Moderate
Chen	2016	13	Tree nuts	Stroke mortality	HL	0.93 (0.77-1.13)	130987 / 2130 (3)	0	Moderate
Chen	2016	13	Tree nuts	Mortality	HL	0.83 (0.77-0.89)	130987 / 36252 (3)	0	Moderate
Chen	2016	13	Tree nuts	Cancer mortality	HL	0.82 (0.76-0.9)	130987 / 14210 (3)	0	Moderate
Chen	2016	13	Peanuts	CVD mortality	HL	0.78 (0.73-0.85)	265252 / 12052 (5)	18	Moderate
Chen	2016	13	Peanuts	CHD mortality	HL	0.76 (0.69-0.82)	265252 / 7025 (5)	0	Moderate
Chen	2016	13	Peanuts	Stroke mortality	HL	0.83 (0.71-0.97)	265252 / 3315 (5)	46	Moderate
Chen	2016	13	Peanuts	Mortality	HL	0.85 (0.81-0.89)	265252 / 44396 (5)	34	Moderate
Chen	2016	13	Peanuts	Cancer mortality	HL	0.93 (0.87-0.99)	265252 / 17742 (5)	19	Moderate
Chen	2016	13	All nuts	CVD mortality	28g/week	0.94 (0.93-0.96)	509871 / 20362 (16)	60	Moderate
Chen	2016	13	All nuts	CHD mortality	28g/week	0.94 (0.93-0.96)	412892 / 10399 (13)	34	Moderate
Chen	2016	13	All nuts	Stroke mortality	28g/week	0.95 (0.91-1)	432352 / 4831 (12)	61	Moderate
Chen	2016	13	All nuts	Mortality	28g/week	0.96 (0.94-0.97)	766470 / 81034 (18)	72	Moderate
Chen	2016	13	All nuts	Cancer mortality	28g/week	0.97 (0.96-0.99)	434405 / 21302 (10)	23	Moderate
Chen	2016	13	Tree nuts	CVD mortality	28g/week	0.91 (0.86-0.95)	130987 / 9456 (3)	0	Moderate
Chen	2016	13	Tree nuts	CHD mortality	28g/week	0.89 (0.84-0.95)	130987 / 6394 (3)	15	Moderate
Chen	2016	13	Tree nuts	Stroke mortality	28g/week	0.96 (0.87-1.06)	130987 / 2130 (3)	0	Moderate
Chen	2016	13	Tree nuts	Mortality	28g/week	0.91 (0.89-0.94)	130987 / 36252 (3)	0	Moderate
Chen	2016	13	Tree nuts	Cancer mortality	28g/week	0.92 (0.88-0.96)	130987 / 14210 (3)	0	Moderate
Chen	2016	13	Peanuts	CVD mortality	28g/week	0.84 (0.77-0.93)	265252 / 12052 (5)	80	Moderate
Chen	2016	13	Peanuts	CHD mortality	28g/week	0.87 (0.81-0.95)	265252 / 7025 (5)	53	Moderate
Chen	2016	13	Peanuts	Stroke mortality	28g/week	0.84 (0.73-0.96)	265252 / 3315 (5)	72	Moderate
Chen	2016	13	Peanuts	Mortality	28g/week	0.91 (0.87-0.95)	265252 / 44396 (5)	64	Moderate
Chen	2016	13	Peanuts	Cancer mortality	28g/week	0.97 (0.93-1.01)	265252 / 17742 (5)	23	Moderate
Grosso	2014	14	All nuts	CVD mortality	HL	0.71 (0.62-0.81)	354933 / 7775 (9)	25	Moderate
Grosso	2014	14	All nuts	Mortality	HL	0.77 (0.69-0.87)	354933 / 44636 (9)	56	Moderate
Grosso	2014	14	All nuts	Cancer mortality	HL	0.86 (0.75-0.98)	354933 / 10423 (9)	32	Moderate
Grosso	2014	14	All nuts	CVD mortality	28g	0.61 (0.42-0.91)	354933 / 7775 (9)	74	Moderate
Grosso	2014	14	All nuts	Mortality	28g	0.73 (0.6-0.88)	354933 / 44636 (9)	53	Moderate

Grosso	2014	14	All nuts	Cancer mortality	28g	0.69 (0.33-1.45)	354933 / 10423 (9)	70	Moderate
Guo	2013	41	All nuts	Diabetes mellitus	HL	0.98 (0.84-1.15)	263663 / 11 580 (6)	68	Moderate
Guo	2013	41	All nuts	Diabetes mellitus	28g	1.04 (0.95-1.14)	263663 / 11 580 (6)	58	Moderate
Li	2017	42	All nuts	Metabolic syndrome	28g/week	0.96 (0.92-0.99)	20666 / 4625 (6)	0	High
Li	2017	42	All nuts	Overweight or obesity-	28g/week	0.97 (0.95-0.98)	203591 / 28678 (6)	0	High
Li	2017	42	All nuts	Obesity	28g/week	0.95 (0.89-1.02)	178633 / 18557 (6)	74	High
Luo	2013	43	All nuts	CVD	28g	0.71 (0.59-0.85)	/ 8862 (4)	49	High
Luo	2013	43	All nuts	CHD	28g	0.72 (0.64-0.81)	/ 6623 (6)	0	High
Luo	2013	43	All nuts	Stroke	28g	0.91 (0.81-1.02)	/ 6487 (5)	20	High
Luo	2013	43	All nuts	Mortality	28g	0.83 (0.76-0.91)	/ 48818 (11)	62	High
Luo	2013	43	All nuts	Diabetes mellitus	28g	0.88 (0.84-0.92)	/ 12655 (8)	68	High
Ma	2014	44	All nuts	CHD	HL	0.66 (0.58-0.75)	347477 / 6127 (13)	40	Low
Ma	2014	44	All nuts	CHD	28g	0.7 (0.58-0.83)	/ 4886 (7)	0	Low
Mayhew	2015	45	All nuts	CVD mortality	HL	0.73 (0.68-0.78)	243795 / 13726 (5)	16	Moderate
Mayhew	2015	45	All nuts	CHD	HL	0.66 (0.48-0.91)	123971 / 4757 (3)	88	Moderate
Mayhew	2015	45	All nuts	Stroke	HL	1.05 (0.69-1.61)	157826 / 4318 (2)	77	Moderate
Mayhew	2015	45	All nuts	Stroke mortality	HL	0.83 (0.69-1)	159322 / 2166 (3)	0	Moderate
Mayhew	2015	45	All nuts	Mortality	HL	0.81 (0.77-0.85)	277432 / 49232 (10)	43	Moderate
Mayhew	2015	45	All nuts	CVD	16g	0.72 (0.55-0.96)	6309 / 634 (1)	0	Moderate
Mayhew	2015	45	All nuts	CVD mortality	16g	0.78 (0.63-1)	243795 / 13726 (5)	16	Moderate
Mayhew	2015	45	All nuts	CHD mortality	16g	0.78 (0.57-1.08)	278584 / 8454 (7)	0	Moderate
Mayhew	2015	45	All nuts	Stroke mortality	16g	0.85 (0.55-1.31)	159322 / 2166 (3)	0	Moderate
Mayhew	2015	45	All nuts	Mortality	16g	0.81 (0.75-0.92)	277432 / 49232 (10)	43	Moderate
Naghshi	2020	33	All nuts	Cancer mortality	HL	0.87 (0.82-0.91)	819851 / 48038 (10)	23	High
Naghshi	2020	33	Tree nuts	Cancer mortality	HL	0.82 (0.76-0.9)	/ (3)	0	High
Naghshi	2020	33	Peanuts	Cancer mortality	HL	0.92 (0.86-0.99)	/ (4)	34	High
Naghshi	2020	33	All nuts	Cancer mortality	5g	0.96 (0.95-0.98)	/ (10)	30	High
Naghshi	2020	33	Peanuts	Cancer mortality	5g	0.97 (0.92-1.02)	/ (4)	49	High
Schwingshackl	2016	46	All nuts	Mortality	HL	0.8 (0.74-0.86)	/ 80204 (16)	84	High
Schwingshackl	2016	46	All nuts	Mortality	28g	0.76 (0.69-0.84)	/ 80204 (16)	82	High
Schwingshackl	2017	47	All nuts	Diabetes mellitus	HL	0.95 (0.85-1.05)	/ 27016 (8)	67	High
Schwingshackl	2017	47	All nuts	Diabetes mellitus	28g	0.89 (0.71-1.12)	/ 27016 (8)	77	High
Shao	2016	48	All nuts	Stroke	HL	0.88 (0.8-0.97)	/ (14)	0	Moderate
Shao	2016	48	All nuts	Stroke mortality	HL	0.81 (0.72-0.91)	/ ()	0	Moderate
Shao	2016	48	All nuts	Stroke	12g	0.86 (0.79-0.94)	/ (11)	0	Moderate
Shi	2014	49	All nuts	Stroke	HL	0.9 (0.81-0.99)	228799 / 5669 (8)	0	Moderate
Shi	2014	49	All nuts	Hemorrhagic stroke incidence	HL	1.17 (0.54-2.54)	210472 / 5601 (3)	78	Moderate

Shi	2014	49	All nuts	Ischemic stroke incidence	HL	0.97 (0.84-1.1)	210472 / 5601 (3)	0	Moderate
Weng	2014	50	All nuts	CHD	HL	0.68 (0.59-0.78)	/ 6302 (14)	63	Moderate
Weng	2014	50	All nuts	CHD	28g/week	0.9 (0.87-0.94)	/ 6127 (13)	68	Moderate
Wu	2013	34	All nuts	Diabetes mellitus	HL	0.98 (0.84-1.14)	/ (5)	74	Low
Zhang	2018	51	All nuts	Metabolic syndrome	HL	0.84 (0.76-0.92)	89224 / (11)	79	Moderate
Zhang	2018	51	Tree nuts	Metabolic syndrome	HL	0.97 (0.94-1)	/ (4)	0	Moderate
Zhang	2018	51	Peanuts	Metabolic syndrome	HL	1.01 (0.96-1.06)	/ (2)	0	Moderate
Zhang	2019	35	All nuts	Cancer mortality	HL	0.9 (0.88-0.92)	/ 49161 (9)	14	High
Zhang	2014	52	All nuts	Stroke	HL	0.9 (0.83-0.98)	476181 / (9)	0	Moderate
Zhang	2014	52	All nuts	Hemorrhagic stroke incidence	HL	1.53 (0.97-2.41)	194307 / 5454 (2)	0	Moderate
Zhang	2014	52	All nuts	Ischemic stroke incidence	HL	0.9 (0.74-1.09)	210472 / 5601 (3)	0	Moderate

* Abbreviations: CHD: coronary heart disease; CVD: cardiovascular disease

Table S4: Summary of data on adverse reactions including allergy and anaphylactic reactions on nut and seed consumption.

First author and title	Main outcome	Search period (end)	Studies (n)	Findings	Conclusion from the study
Conrado. Global patterns in anaphylaxis due to specific foods: A systematic review	Prevalence of anaphylaxis and allergy to peanut and tree nuts	2020 (Nov)	65	The estimated prevalence estimates (in %) among adults were as follows: Peanut allergy: Europe 0.35 (0.20-0.60), North America/Southwest Pacific 1.8 (0.6-1.9), Asia 0.46 (0.36-0.58); Tree nuts: North America/Southwest Pacific 1.2 (1.1-1.3); Cashew nuts: North America/Southwest Pacific 0.5 (0.5-0.6); Hazelnut: Europe 0.86 (0.39-1.90), North America/Southwest Pacific 0.6 (0.5-0.7); Walnut: Europe 0.30 (0.14-0.66), North America/Southwest Pacific 0.6 (0.6-0.7); Sesame: Europe 0.01 (0-1.35), North America/Southwest Pacific 0.2 (0.2-0.3). The estimated prevalence estimates (in %) among children were as follows: Peanut: Europe 0.42 (0.25-0.70), North America/Southwest Pacific 2.60 (2.17-3.11), Asia 0.21 (0.17-0.27); Tree nuts: North America/Southwest Pacific 1.77 (1.26-2.47), Asia 0.12 (0-6.63); Cashew nuts: Europe 0.11 (0-0.62), North America/Southwest Pacific 1.04 (0.59-1.84); Hazelnut: Europe 0.28 (0.10-0.77), North America/Southwest Pacific 0.61 (0.54-0.69); Walnut: Europe 0.12 (0.04-0.41), North America/Southwest Pacific 0.61 (0.54-0.69); Sesame: Europe 0.07 (0.01-0.88), North America/Southwest Pacific 0.21 (0.17-0.25), Asia 0.07 (0.05-0.10).	The estimated prevalence of allergy to different types of nuts varied across geographic regions, as well as between children and adults. Local legislation for allergen disclosure generally reflects those allergens commonly responsible for food anaphylaxis
McWilliam. The Prevalence of Tree Nut Allergy: A Systematic Review	Tree nut allergy	2014 (Dec)	36 (24 in children, 18 from Europe, 5 in USA)	Challenge-confirmed IgE-mediated tree nut allergy prevalence was less than 2 % (although only seven studies used this gold standard) while probable tree nut allergy prevalence ranged from 0.05-4.9%. Prevalence estimates that included oral allergy syndrome (OAS) reactions to tree nut were significantly higher (8–11.4%) and were predominantly from Europe. Prevalence of individual tree nut allergies varied significantly by region with hazelnut the most common tree nut allergy in Europe, walnut and cashew in the USA and Brazil nut, almond, and walnut most reported in the UK.	Prevalence of individual tree nut allergies varied significantly by region with hazelnut the most common tree nut allergy in Europe, walnut and cashew in the USA and Brazil nut, almond and walnut most reported in the UK

Nwaru. Prevalence of common food allergies in Europe: a systematic review and meta-analysis	Food allergies including peanut and tree nuts	2012 (Sep)	50 (42 in meta-analyses)	The overall pooled estimates for all age groups of self-reported lifetime prevalence of allergy to peanut was 0.4% (0.3–0.6) and 1.3% (1.2–1.5) for tree nuts. The prevalence of food-challenge-defined allergy to peanut was 0.2% (0.2–0.3) and correspondingly 0.5% (0.08–0.8) for tree nuts. Significant heterogeneity between the studies. Peanut allergy: The overall lifetime prevalence of self-reported peanut allergy was 0.4% (95% CI 0.3–0.6), 1.7% (95% CI 1.5–1.8) for point self-reported prevalence, 1.7% (95% CI 1.6–1.9) for SPT positivity, 8.6% (95% CI 8.2–9.0) for specific IgE-positivity, 0.2% (95% CI for 0.2–0.3) for FC positivity, and 1.6% (95% CI 1.2–1.9) for FC or history of peanut allergy. In most cases, the estimates were higher in older age groups than in younger children, while the region-stratified estimates were mostly higher in Western Europe than in other regions. The overall lifetime prevalence of self-reported tree nut allergy was 1.3% (95% CI 1.2–1.5), 1.8% (95% CI 1.6–2.0) for point self-reported prevalence, 0.6% (95% CI 0.5–0.7) for SPT positivity, 0.5% (95% CI for 0.08–0.8) for FC positivity, and 0.1% (95% CI 0.1–0.2) for FC or history of tree nut allergy. The estimates were higher in older age groups than in younger children, while the region-stratified estimates were mostly higher in Northern Europe than in other regions.	The lifetime self-reported prevalence of allergy to common foods in Europe ranged from 0.1 to 6.0%. The heterogeneity between studies was high, and participation rates varied across studies reaching as low as <20% in some studies.
Patel. Hypersensitivities to sesame and other common edible seeds	Hypersensitivities to sesame and other seeds	2016 (Mar)		Prevalence of sesame allergy was estimated at 0.1–0.2%. The estimate in United States, through a telephone survey, is 0.1%. In Israel, sesame allergy was reported as the third most common cause of IgE-mediated food reactions and as the second most common cause of food-induced anaphylaxis. Reported cases comprised all ages, including early infancy. In a pediatric series, the median age was 1 year 5. In a study from Saudi Arabia, sesame was the third most common trigger of food-induced anaphylaxis. Study in Italy of 351 adults sensitized to various fruits and vegetable, there were five with systemic symptoms to seeds (sesame and sunflower). Among 1110 adults with food allergy in general, sesame and sunflower were implicated in six cases, including two had anaphylaxis.	Allergy to sesame was more reported than to other seeds, with an estimated prevalence of 0.1–0.2%.

Patel. Using data from food challenges to inform management of consumers with food allergy: A systematic review with individual participant data meta-analysis	Food allergies and rate of anaphylaxis to low-level peanut exposure and reproducibility of reaction thresholds	2020 (Sep)	19 (3151 participants, 534 underwent further peanut challenge)	At individual participant data meta-analysis, 4.5% (95%CI 1.9%-10.1%) of individuals reacted to 5 mg or less of peanut protein with anaphylaxis (moderate heterogeneity [I2 = 57%]). Intraindividual thresholds varied. In all, 2.4% (95% CI, 1.1% to 5.0%) of patients initially tolerated 5 mg of peanut protein but then reacted to this dose at subsequent challenge (low heterogeneity [I2 = 16%]); none developed anaphylaxis. In all, 12 studies included an initial challenge dose of 1 mg or less of peanut protein; at meta-analysis, 4.2% (95% CI, 0.7% to 22.3%) of reactions to 1 mg or less of peanut protein would be anaphylaxis (moderate heterogeneity [I2 = 56%]). Because of fewer reactions at this level of allergen exposure, no sensitivity analyses were undertaken. Around 5% of individuals reacting to the eliciting dose of 1.5 mg of peanut protein (5 mg peanut) level of exposure to peanut might develop anaphylaxis in response to that dose.	Among individuals with peanut allergy, 1 to 6 anaphylaxis events are estimated per 2500 patients exposed to low-dose nuts protein
Rona. The prevalence of food allergy: A meta-analysis	Prevalence of food allergies including peanuts	2005 (Dec)	15 self report), 3 (IgE), 6 (skin prick test), 8 (self report+sensitization), 3 (food challenge)	Self-reported prevalence of food allergy varied from 0% to 2% for peanuts. The prevalence of symptomatic and sensitized (skin prick test or IgE) ranged from 0.5-2.5% for peanuts. Prevalence of IgE sensitization ranges from less than 1% to 6%, equivalent figures for skin prick test were 1% to 6%. The estimated prevalence of allergy based on food challenge varied between 0.4% to 1.6% in different studies (3 studies in total).	There is a marked heterogeneity in the prevalence of food allergy due to differences in study design, methodology, and populations
van der Valk. Systematic review on cashew nut allergy	Cashew nut allergy	2013 (?)	7	0.08% of children under 4 in the United Kingdom were sensitized to cashew nuts. Thirty per cent of the peanut and 74% of the cashew nut sensitized patients developed an anaphylactic reaction after ingestion	Among anaphylaxis reactions, cashew nut allergy is a relatively common cause. Avoidance of pistachio nuts must currently be advised in case of a cashew nut allergy.
Versluis. Frequency, severity and causes of unexpected allergic reactions to food: a systematic literature review	Food allergies (various)	2013 (Apr)	13	Among 530 reactions in Spain, 9% of reactions due to nuts, 32% were anaphylactic (none fatal). US: Registry data from the United States estimating ca. 5 fatalities/year due to nuts (3/4 of these due to peanuts). Severe reaction not uncommon, but life-threatening reactions are rare.	Anaphylaxis reactions are rare, but among these peanuts seems to be leading food allergen

Zuidmeer. The prevalence of plant food allergies: a systematic review	Prevalence of food allergies to tree nuts/all nuts	2006 (Dec)	17 (6 included food challenge)	Prevalence of perceived allergies to tree nuts in following ages as follows: 0-6 years of 0.03% to 0.2%, 6-18 years of 0.2% to 2.3%, and adults of 0.4% to 1.4%. The prevalence of perceived reactions to any nuts ranged from 0% to 7.3%. Only 6 studies included food challenge tests with prevalence of allergy ranging from 0.1% to 4.3% for tree nuts.	Population-based prevalence estimates for allergies to plant products determined by the diagnostic gold standard are scarce. There was considerable heterogeneity in the prevalence estimates of sensitization or perceived allergic reactions to plant food.

Supplementary figure 1: Summary of associations from the most recent meta-analyses between high compared to low consumption of nuts and risk of various morbidities and mortalities. Reference number is listed in brackets and search year is listed within the parentheses.

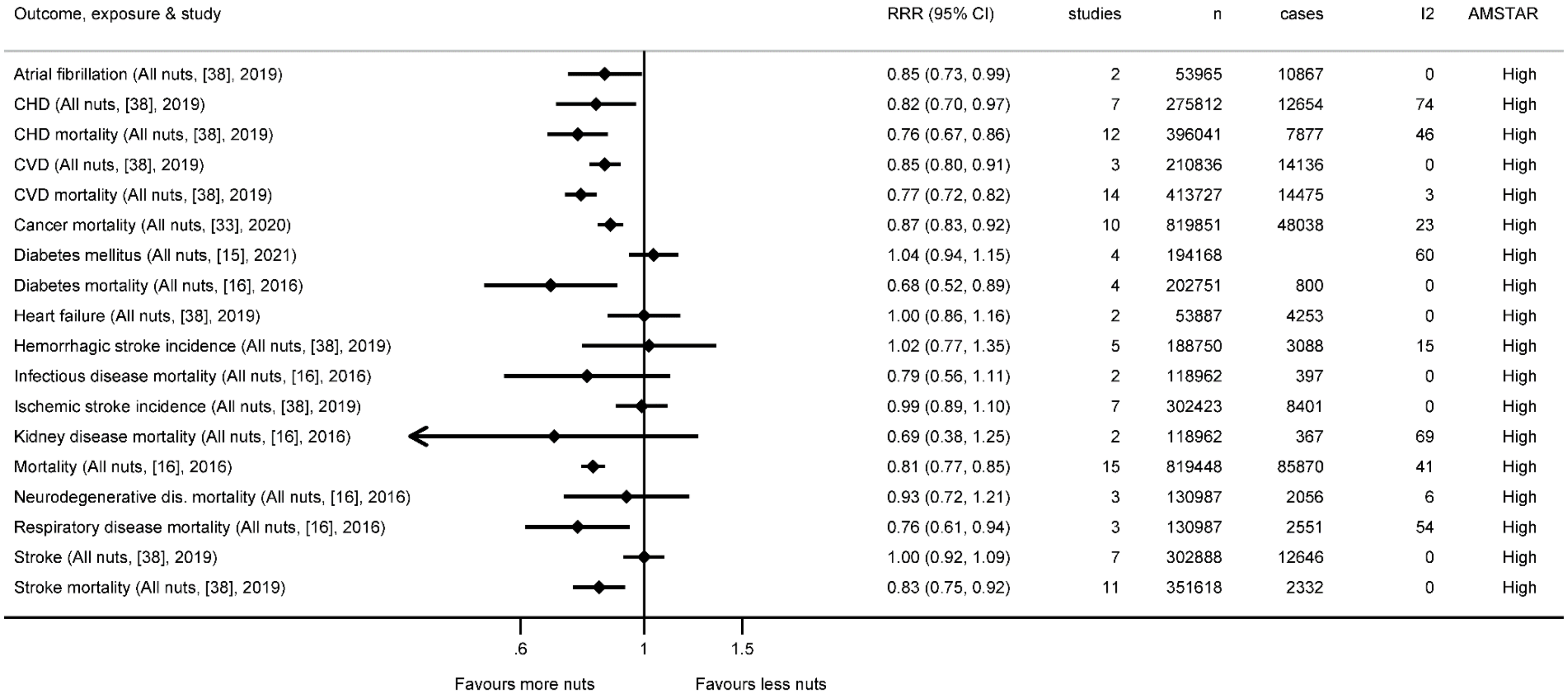

* Abbreviations: CHD: coronary heart disease; CVD: cardiovascular disease

Supplementary figure 2: Summary of per serving associations from the most recent meta-analyses between consumption of 28g nuts and risk of various morbidities and mortalities. Reference number is listed in brackets and search year is listed within the parentheses.

Outcome, exposure & study	RRR (95% CI)	studies	n	cases	I2	AMSTAR
CHD (All nuts, [38], 2019)	0.75 (0.64, 0.88)	7	275812	12654	74	High
CHD mortality (All nuts, [38], 2019)	0.67 (0.52, 0.87)	12	396041	7877	46	High
CVD (All nuts, [38], 2019)	0.87 (0.81, 0.93)	3	210836	14136	0	High
CVD mortality (All nuts, [38], 2019)	0.71 (0.61, 0.83)	14	413727	14475	3	High
Cancer mortality (All nuts, [33], 2020)	0.85 (0.80, 0.90)	10			30	High
Diabetes mellitus (All nuts, [47], 2017)	0.89 (0.71, 1.12)	8		27016	77	High
Diabetes mortality (All nuts, [16], 2016)	0.61 (0.43, 0.87)	4	202751	800	0	High
Heart failure (All nuts, [39], 2017)	1.09 (0.97, 1.22)	2		3613	0	High
Hemorrhagic stroke incidence (All nuts, [38], 2019)	1.05 (0.77, 1.43)	5	188750	3088	15	High
Infectious disease mortality (All nuts, [16], 2016)	0.25 (0.07, 0.87)	2	118962	397	54	High
Ischemic stroke incidence (All nuts, [38], 2019)	1.06 (0.86, 1.31)	7	302423	8401	0	High
Kidney disease mortality (All nuts, [16], 2016)	0.27 (0.04, 1.87)	2	118962	367	61	High
Metabolic syndrome (All nuts, [42], 2017)	0.96 (0.93, 1.00)	6	20666	4625	0	High
Mortality (All nuts, [16], 2016)	0.78 (0.72, 0.84)	16	819448	85870	66	High
Neurodegenerative dis. mortality (All nuts, [16], 2016)	0.65 (0.40, 1.07)	3	130987	2056	6	High
Obesity (All nuts, [42], 2017)	0.95 (0.89, 1.02)	6	178633	18557	74	High
Respiratory disease mortality (All nuts, [16], 2016)	0.48 (0.26, 0.89)	3	130987	2551	61	High
Stroke (All nuts, [38], 2019)	1.06 (0.97, 1.15)	7	302888	12646	0	High
Stroke mortality (All nuts, [38], 2019)	1.01 (0.87, 1.17)	11	351618	2332	0	High

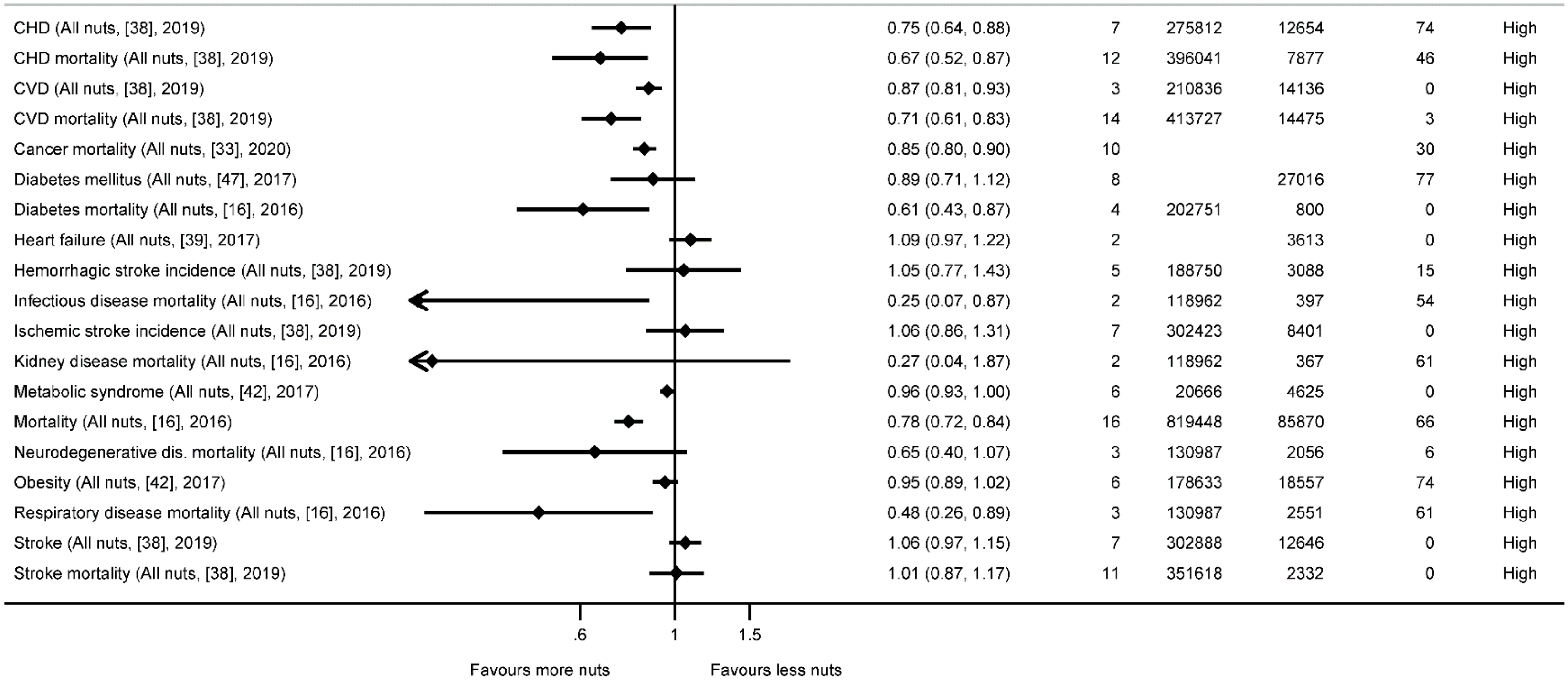

* Abbreviations: CHD: coronary heart disease; CVD: cardiovascular disease

Supplementary figure 3: Summary of associations from meta-analyses between high compared to low consumption of nuts and risk of various cardiovascular morbidities and mortalities. Reference number is listed in brackets and search year is listed within the parentheses. Number of studies, participants (n) and cases, as well as heterogeneity (I^2) is also listed in the right column.

Outcome, exposure & study	RRR (95% CI)	studies	n	cases	I2
Atrial fibrillation (All nuts, [29], 2019)	0.85 (0.73, 0.99)	2	53965	10867	0
CHD (All nuts, [29], 2019)	0.82 (0.70, 0.97)	7	275812	12654	74
CHD (All nuts, [30], 2017)	0.80 (0.62, 1.03)	4		5480	79
CHD (All nuts, [15], 2016)	0.76 (0.69, 0.84)	11	315397	12331	48
CHD (All nuts, [37], 2015)	0.66 (0.48, 0.91)	3	123971	4757	88
CHD (All nuts, [36], 2014)	0.66 (0.58, 0.75)	13	347477	6127	40
CHD (All nuts, [43], 2014)	0.68 (0.59, 0.78)	14		6302	63
CHD (Peanuts, [29], 2019)	0.85 (0.79, 0.92)	3	210836	8390	0
CHD (Peanuts, [15], 2016)	0.76 (0.70, 0.83)	5	265252	7025	0
CHD (Tree nuts, [29], 2019)	0.77 (0.70, 0.84)	3	210836	8390	61
CHD (Tree nuts, [15], 2016)	0.79 (0.68, 0.92)	3	130987	6394	28
CHD (Walnuts, [29], 2019)	0.79 (0.66, 0.94)	3	144021	2685	4
CHD mortality (All nuts, [29], 2019)	0.76 (0.67, 0.86)	12	396041	7877	46
CHD mortality (All nuts, [12], 2016)	0.73 (0.67, 0.80)	13	429833	10083	14
CHD mortality (Peanuts, [29], 2019)	0.75 (0.64, 0.88)	2	134265	2119	0
CHD mortality (Peanuts, [12], 2016)	0.76 (0.70, 0.83)	5	265252	7025	0
CHD mortality (Tree nuts, [12], 2016)	0.79 (0.68, 0.92)	3	130987	6394	28
CVD (All nuts, [29], 2019)	0.85 (0.80, 0.91)	3	210836	14136	0
CVD (All nuts, [15], 2016)	0.81 (0.74, 0.89)	11	376228	18655	52
CVD (Peanuts, [29], 2019)	0.87 (0.81, 0.93)	3	210836	14136	0
CVD (Peanuts, [15], 2016)	0.81 (0.75, 0.87)	5	265252	12043	15
CVD (Tree nuts, [29], 2019)	0.85 (0.79, 0.91)	3	210836	14136	0
CVD (Tree nuts, [15], 2016)	0.81 (0.74, 0.89)	3	130987	9456	0
CVD (Walnuts, [29], 2019)	0.81 (0.71, 0.92)	3	144021	5255	3
CVD mortality (All nuts, [29], 2019)	0.77 (0.72, 0.82)	14	413727	14475	3
CVD mortality (All nuts, [12], 2016)	0.75 (0.71, 0.79)	16	524610	19574	0
CVD mortality (All nuts, [37], 2015)	0.73 (0.68, 0.78)	5	243795	13726	16
CVD mortality (All nuts, [13], 2014)	0.71 (0.62, 0.81)	9	354933	7775	25
CVD mortality (Peanuts, [29], 2019)	0.77 (0.70, 0.85)	2	134265	5572	0
CVD mortality (Peanuts, [12], 2016)	0.78 (0.72, 0.84)	5	265252	12052	18
CVD mortality (Tree nuts, [12], 2016)	0.81 (0.74, 0.89)	3	130987	9456	0
Heart failure (All nuts, [29], 2019)	1.00 (0.86, 1.16)	2	53887	4253	0
Heart failure (All nuts, [30], 2017)	0.99 (0.86, 1.14)	3		3613	57
Hemorrhagic stroke incidence (All nuts, [29], 2019)	1.02 (0.77, 1.35)	5	188750	3088	15
Hemorrhagic stroke incidence (All nuts, [47], 2014)	1.53 (0.97, 2.41)	2	194307	5454	0
Hemorrhagic stroke incidence (All nuts, [42], 2014)	1.17 (0.54, 2.54)	3	210472	5601	78
Ischemic stroke incidence (All nuts, [29], 2019)	0.99 (0.89, 1.10)	7	302423	8401	0
Ischemic stroke incidence (All nuts, [47], 2014)	0.90 (0.74, 1.09)	3	210472	5601	0
Ischemic stroke incidence (All nuts, [42], 2014)	0.97 (0.85, 1.11)	3	210472	5601	0
Stroke (All nuts, [29], 2019)	1.00 (0.92, 1.09)	7	302888	12646	0
Stroke (All nuts, [30], 2017)	0.94 (0.85, 1.04)	6		7490	18
Stroke (All nuts, [15], 2016)	0.89 (0.82, 0.97)	10	396768	9272	0
Stroke (All nuts, [41], 2016)	0.88 (0.80, 0.97)	14			0
Stroke (All nuts, [37], 2015)	1.05 (0.69, 1.60)	2	157826	4318	77
Stroke (All nuts, [47], 2014)	0.90 (0.83, 0.98)	9	476181		0
Stroke (All nuts, [42], 2014)	0.90 (0.81, 0.99)	8	228799	5669	0
Stroke (Peanuts, [29], 2019)	0.90 (0.81, 0.99)	3	210836	5910	13
Stroke (Peanuts, [15], 2016)	0.83 (0.69, 1.00)	5	265252	3315	46
Stroke (Tree nuts, [29], 2019)	1.00 (0.90, 1.12)	3	210836	5910	0
Stroke (Tree nuts, [15], 2016)	0.89 (0.82, 0.97)	3	130987	2130	0
Stroke (Walnuts, [29], 2019)	0.85 (0.71, 1.02)	3	144021	5910	19
Stroke mortality (All nuts, [29], 2019)	0.83 (0.75, 0.92)	11	351618	2332	0
Stroke mortality (All nuts, [12], 2016)	0.82 (0.73, 0.92)	12	449293	4398	0
Stroke mortality (All nuts, [41], 2016)	0.81 (0.72, 0.91)				0
Stroke mortality (All nuts, [37], 2015)	0.83 (0.69, 1.00)	3	159322	2166	0
Stroke mortality (Peanuts, [29], 2019)	0.83 (0.73, 0.95)	4	253227	3036	57
Stroke mortality (Peanuts, [12], 2016)	0.83 (0.71, 0.97)	5	265252	3315	46
Stroke mortality (Tree nuts, [29], 2019)	0.93 (0.77, 1.13)	3	118962	1851	0
Stroke mortality (Tree nuts, [12], 2016)	0.93 (0.77, 1.13)	3	130987	2130	0

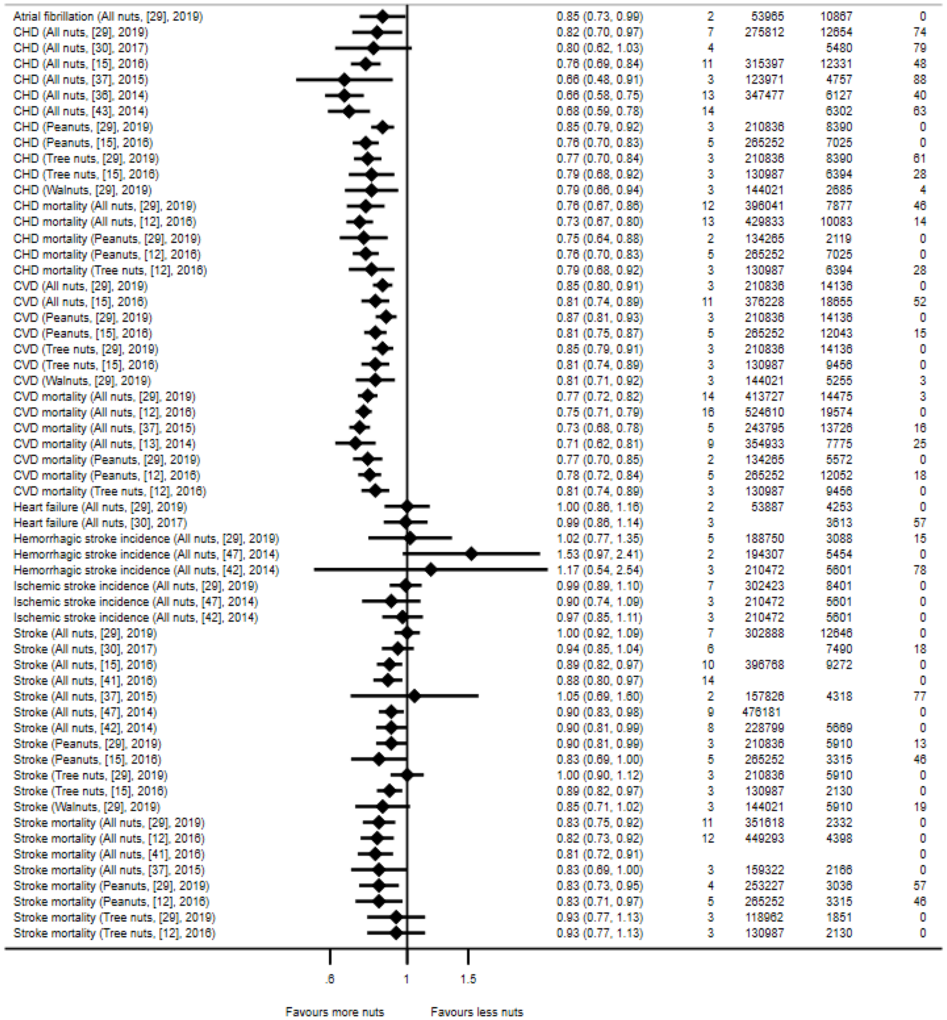

* Abbreviations: CHD: coronary heart disease; CVD: cardiovascular disease

Supplementary figure 4: Summary of associations from meta-analyses between per serving of 28g consumption of nuts and risk of various cardiovascular morbidities and mortalities. Reference number is listed in brackets and search year is listed within the parentheses. Number of studies, participants (n) and cases, as well as heterogeneity (I^2) is also listed in the right column.

Outcome, exposure & study	RRR (95% CI)	studies	n	cases	I2
CHD (All nuts, [38], 2019)	0.75 (0.64, 0.88)	7	275812	12654	74
CHD (All nuts, [39], 2017)	0.67 (0.43, 1.05)	4		5480	85
CHD (All nuts, [16], 2016)	0.71 (0.63, 0.80)	11	315397	12331	47
CHD (All nuts, [50], 2014)	0.90 (0.87, 0.94)	13		6127	68
CHD (All nuts, [44], 2014)	0.70 (0.59, 0.84)	7		4886	0
CHD (All nuts, [37], 2013)	0.65 (0.49, 0.85)	4	141390	2101	0
CHD (All nuts, [43], 2013)	0.72 (0.64, 0.81)	6		6623	0
CHD (Peanuts, [16], 2016)	0.69 (0.57, 0.84)	5	265252	7025	45
CHD (Tree nuts, [16], 2016)	0.73 (0.63, 0.85)	3	130987	6394	0
CHD mortality (All nuts, [38], 2019)	0.67 (0.52, 0.87)	12	396041	7877	46
CHD mortality (All nuts, [13], 2016)	0.94 (0.93, 0.96)	13	412892	10399	34
CHD mortality (All nuts, [45], 2015)	0.65 (0.37, 1.13)	7	278584	8454	0
CHD mortality (All nuts, [37], 2013)	0.62 (0.52, 0.73)	4	206114	6749	28
CHD mortality (Peanuts, [13], 2016)	0.87 (0.80, 0.94)	5	265252	7025	53
CHD mortality (Tree nuts, [13], 2016)	0.89 (0.84, 0.95)	3	130987	6394	15
CVD (All nuts, [38], 2019)	0.87 (0.81, 0.93)	3	210836	14136	0
CVD (All nuts, [16], 2016)	0.79 (0.70, 0.89)	12	376228	18655	60
CVD (All nuts, [45], 2015)	0.56 (0.35, 0.92)	1	6309	634	0
CVD (All nuts, [43], 2013)	0.71 (0.59, 0.85)	4		8862	49
CVD (Peanuts, [16], 2016)	0.64 (0.50, 0.81)	5	265252	12043	77
CVD (Tree nuts, [16], 2016)	0.75 (0.67, 0.84)	3	130987	9456	0
CVD mortality (All nuts, [38], 2019)	0.71 (0.61, 0.83)	14	413727	14475	3
CVD mortality (All nuts, [13], 2016)	0.94 (0.93, 0.96)	16	509871	20362	60
CVD mortality (All nuts, [45], 2015)	0.65 (0.43, 0.97)	5	243795	13726	16
CVD mortality (All nuts, [14], 2014)	0.61 (0.41, 0.90)	9	354933	7775	74
CVD mortality (Peanuts, [13], 2016)	0.84 (0.76, 0.92)	5	265252	12052	80
CVD mortality (Tree nuts, [13], 2016)	0.91 (0.87, 0.96)	3	130987	9456	0
Heart failure (All nuts, [39], 2017)	1.09 (0.97, 1.22)	2		3613	0
Hemorrhagic stroke incidence (All nuts, [38], 2019)	1.05 (0.77, 1.43)	5	188750	3088	15
Ischemic stroke incidence (All nuts, [38], 2019)	1.06 (0.86, 1.31)	7	302423	8401	0
Stroke (All nuts, [38], 2019)	1.06 (0.97, 1.15)	7	302888	12646	0
Stroke (All nuts, [39], 2017)	0.99 (0.84, 1.17)	6		7490	45
Stroke (All nuts, [48], 2016)	0.74 (0.62, 0.88)	11			0
Stroke (All nuts, [16], 2016)	0.93 (0.83, 1.05)	11	396768	9272	14
Stroke (All nuts, [43], 2013)	0.91 (0.81, 1.02)	5		6487	20
Stroke (All nuts, [37], 2013)	0.82 (0.60, 1.11)	4	155685	5544	73
Stroke (Peanuts, [16], 2016)	0.63 (0.41, 0.96)	5	265252	3315	78
Stroke (Tree nuts, [16], 2016)	0.89 (0.69, 1.14)	3	130987	2130	0
Stroke mortality (All nuts, [38], 2019)	1.01 (0.87, 1.17)	11	351618	2332	0
Stroke mortality (All nuts, [13], 2016)	0.95 (0.91, 1.00)	12	432352	4831	61
Stroke mortality (All nuts, [45], 2015)	0.75 (0.35, 1.61)	3	159322	2166	0
Stroke mortality (Peanuts, [13], 2016)	0.84 (0.73, 0.96)	5	265252	3315	72
Stroke mortality (Tree nuts, [13], 2016)	0.96 (0.87, 1.06)	3	130987	2130	0

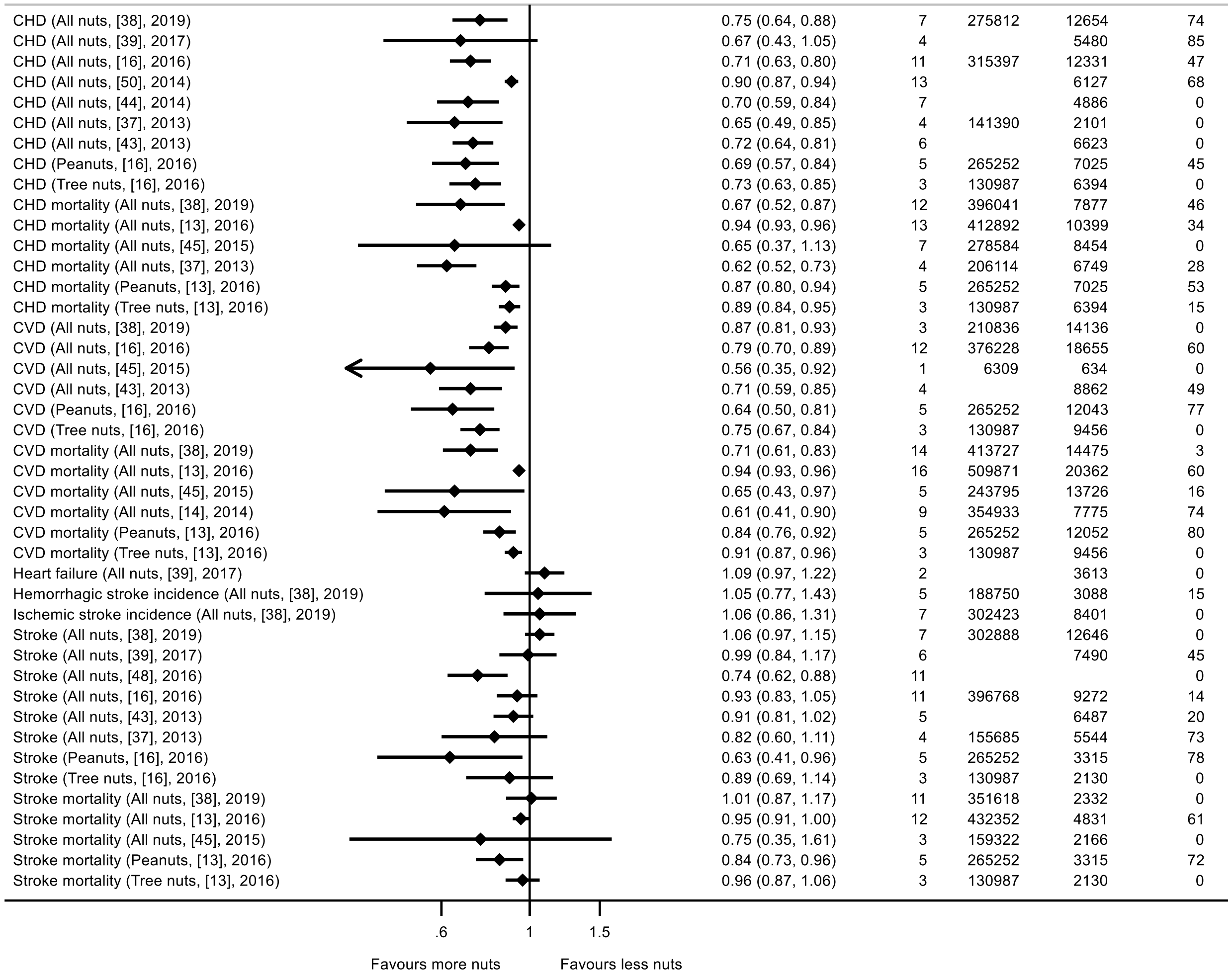

* Abbreviations: CHD: coronary heart disease; CVD: cardiovascular disease

Supplementary figure 5: Summary of associations from meta-analyses between high compared to low consumption of nuts and risk of mortality. Reference number is listed in brackets and search year is listed within the parentheses. Number of studies, participants (n) and cases, as well as heterogeneity (I^2) is also listed in the right column.

Outcome, exposure & study	RRR (95% CI)	studies	n	cases	I2
Mortality (All nuts, [16], 2016)	0.81 (0.77, 0.85)	15	819448	85870	41
Mortality (All nuts, [13], 2016)	0.81 (0.78, 0.84)	16	498730	66568	22
Mortality (All nuts, [46], 2016)	0.80 (0.74, 0.86)	16		80204	84
Mortality (All nuts, [45], 2015)	0.81 (0.77, 0.85)	10	277432	49232	43
Mortality (All nuts, [14], 2014)	0.77 (0.69, 0.86)	9	354933	44636	56
Mortality (Peanuts, [16], 2016)	0.85 (0.82, 0.89)	5	265252	44396	18
Mortality (Peanuts, [13], 2016)	0.85 (0.81, 0.89)	5	265252	44396	34
Mortality (Tree nuts, [13], 2016)	0.83 (0.77, 0.89)	3	130987	36252	0
Mortality (Tree nuts, [16], 2016)	0.80 (0.74, 0.86)	4	202751	42508	58

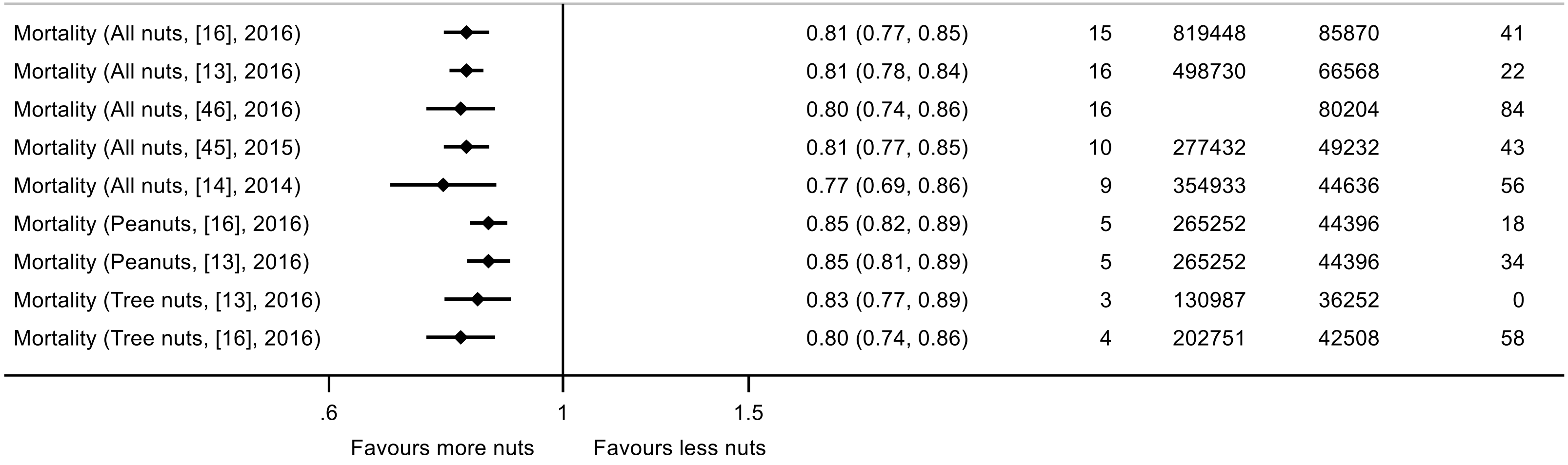

Supplementary figure 6: Summary of associations from meta-analyses between per serving of 28g consumption of nuts and risk of mortality. Reference number is listed in brackets and search year is listed within the parentheses. Number of studies, participants (n) and cases, as well as heterogeneity (I^2) is also listed in the right column.

Outcome, exposure & study	RRR (95% CI)	studies	n	cases	I2
Mortality (All nuts, [16], 2016)	0.78 (0.72, 0.84)	16	819448	85870	66
Mortality (All nuts, [46], 2016)	0.76 (0.69, 0.84)	16		80204	82
Mortality (All nuts, [13], 2016)	0.96 (0.95, 0.98)	18	766470	81034	72
Mortality (All nuts, [45], 2015)	0.69 (0.58, 0.83)	10	277432	49232	43
Mortality (All nuts, [14], 2014)	0.73 (0.60, 0.88)	9	354933	44636	53
Mortality (All nuts, [43], 2013)	0.83 (0.76, 0.91)	11		48818	62
Mortality (Peanuts, [16], 2016)	0.77 (0.69, 0.86)	5	265252	44396	64
Mortality (Peanuts, [13], 2016)	0.91 (0.87, 0.95)	5	265252	44396	64
Mortality (Tree nuts, [16], 2016)	0.82 (0.75, 0.90)	4	202751	42508	70
Mortality (Tree nuts, [13], 2016)	0.91 (0.89, 0.94)	3	130987	36252	0

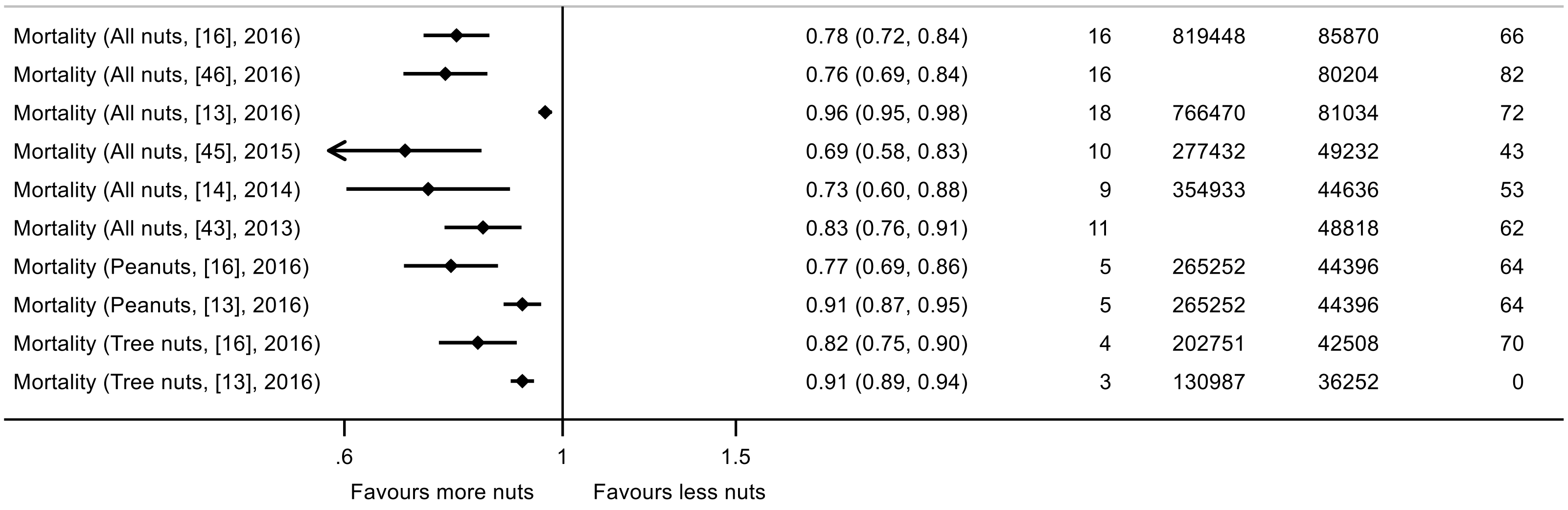

Supplementary figure 7: Summary of associations from meta-analyses between high compared to low consumption of nuts and risk of cancer and cancer-related mortality. Reference number is listed in brackets and search year is listed within the parentheses. Number of studies, participants (n) and cases, as well as heterogeneity (I^2) is also listed in the right column.

Outcome, exposure & study	RRR (95% CI)	studies	n	cases	I2
Cancer mortality (All nuts, [33], 2020)	0.87 (0.83, 0.92)	10	819851	48038	23
Cancer mortality (All nuts, [35], 2019)	0.90 (0.88, 0.92)	9		49161	14
Cancer mortality (All nuts, [16], 2016)	0.82 (0.75, 0.90)	8	254240	17603	28
Cancer mortality (All nuts, [13], 2016)	0.87 (0.81, 0.94)	11	451589	21353	26
Cancer mortality (All nuts, [14], 2014)	0.86 (0.75, 0.98)	9	354933	10423	32
Cancer mortality (All nuts, [40], 2014)	0.85 (0.77, 0.93)	4	247030	14340	17
Cancer mortality (Peanuts, [33], 2020)	0.92 (0.86, 0.99)	4			34
Cancer mortality (Peanuts, [16], 2016)	0.93 (0.87, 0.99)	5	265252	17742	19
Cancer mortality (Peanuts, [13], 2016)	0.93 (0.87, 0.99)	5	265252	17742	19
Cancer mortality (Tree nuts, [33], 2020)	0.82 (0.75, 0.89)	3			0
Cancer mortality (Tree nuts, [13], 2016)	0.82 (0.75, 0.89)	3	130987	14210	0
Cancer mortality (Tree nuts, [16], 2016)	0.82 (0.75, 0.89)	3	130987	14210	0

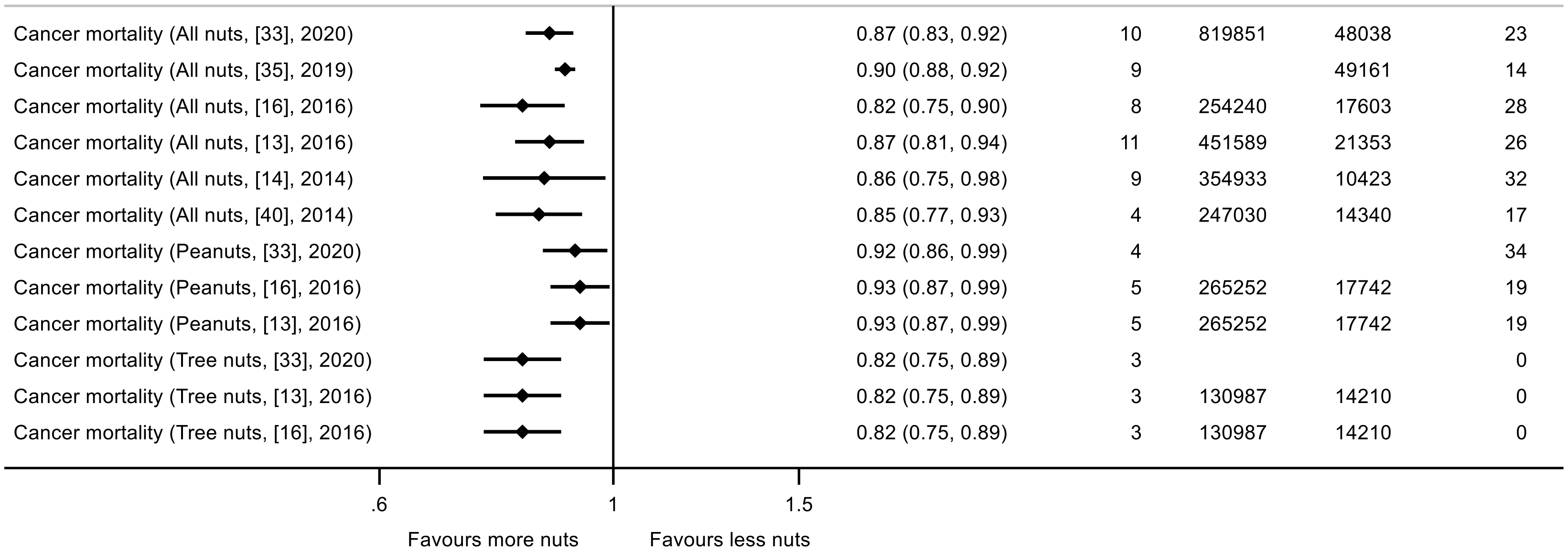

Supplementary figure 8: Summary of associations from meta-analyses between per serving of 28g consumption of nuts and risk of cancer and cancer-related mortality. Reference number is listed in brackets and search year is listed within the parentheses. Number of studies, participants (n) and cases, as well as heterogeneity (I^2) is also listed in the right column.

Outcome, exposure & study	RRR (95% CI)	studies	n	cases	I2
Cancer mortality (All nuts, [33], 2020)	0.85 (0.80, 0.90)	10			30
Cancer mortality (All nuts, [16], 2016)	0.85 (0.76, 0.95)	8	254240	17603	42
Cancer mortality (All nuts, [13], 2016)	0.97 (0.96, 0.99)	10	434405	21302	23
Cancer mortality (All nuts, [14], 2014)	0.69 (0.33, 1.45)	9	354933	10423	70
Cancer mortality (Peanuts, [33], 2020)	0.89 (0.72, 1.09)	4			49
Cancer mortality (Peanuts, [13], 2016)	0.97 (0.93, 1.01)	5	265252	17742	23
Cancer mortality (Peanuts, [16], 2016)	0.92 (0.82, 1.03)	5	265252	17742	30
Cancer mortality (Tree nuts, [13], 2016)	0.92 (0.88, 0.96)	3	130987	14210	0
Cancer mortality (Tree nuts, [16], 2016)	0.80 (0.72, 0.89)	3	130987	14210	0

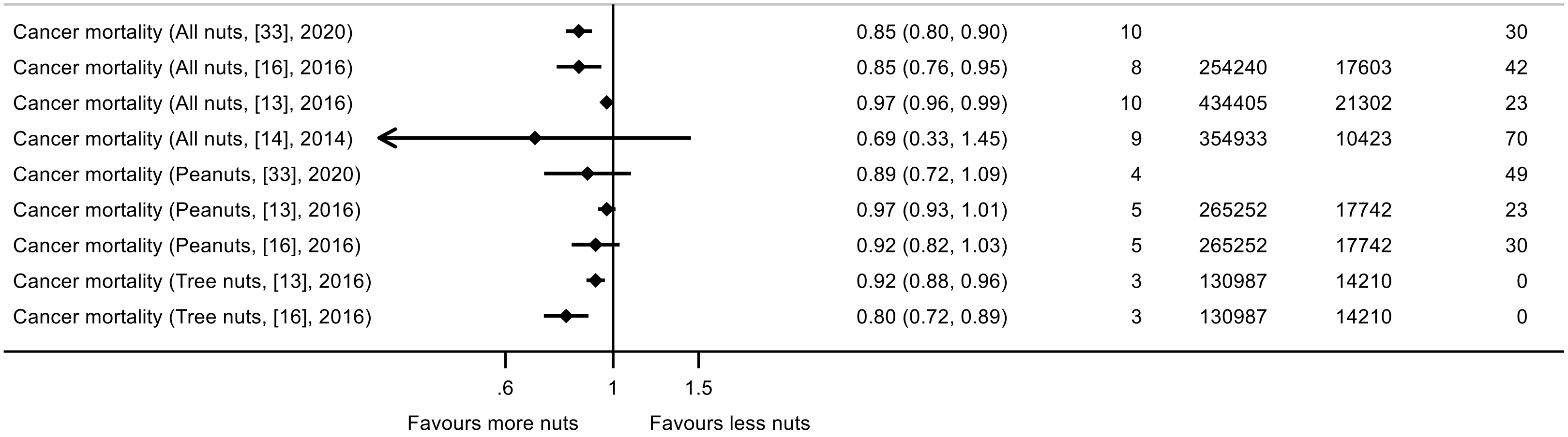

Supplementary figure 9: Summary of associations from meta-analyses between high compared to low consumption of nuts and risk of various diabetes-related morbidities and mortalities. Reference number is listed in brackets and search year is listed within the parentheses. Number of studies, participants (n) and cases, as well as heterogeneity (I^2) is also listed in the right column.

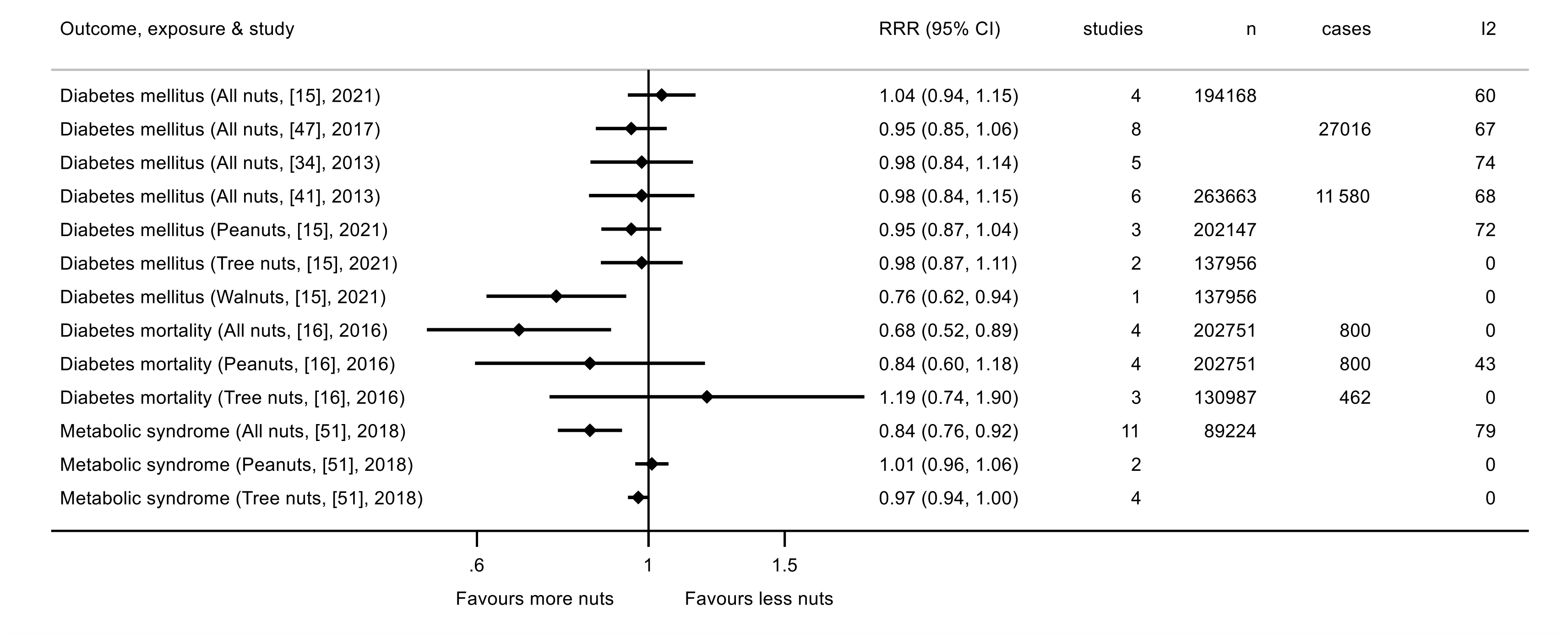

Supplementary figure 10: Summary of associations from meta-analyses between per serving of 28g consumption of nuts and risk of various diabetes-related morbidities and mortalities. Reference number is listed in brackets and search year is listed within the parentheses. Number of studies, participants (n) and cases, as well as heterogeneity (I^2) is also listed in the right column.

Outcome, exposure & study	RRR (95% CI)	studies	n	cases	I2
Diabetes mellitus (All nuts, [47], 2017)	0.89 (0.71, 1.12)	8		27016	77
Diabetes mellitus (All nuts, [37], 2013)	0.78 (0.69, 0.89)	6	230216	13308	22
Diabetes mellitus (All nuts, [43], 2013)	0.88 (0.84, 0.92)	8		12655	68
Diabetes mellitus (All nuts, [41], 2013)	1.04 (0.95, 1.14)	6	263663	11 580	58
Diabetes mortality (All nuts, [16], 2016)	0.61 (0.43, 0.87)	4	202751	800	0
Diabetes mortality (Peanuts, [16], 2016)	0.73 (0.45, 1.19)	4	202751	800	15
Diabetes mortality (Tree nuts, [16], 2016)	1.23 (0.68, 2.24)	3	130987	462	0
Metabolic syndrome (All nuts, [42], 2017)	0.96 (0.93, 1.00)	6	20666	4625	0
Obesity (All nuts, [42], 2017)	0.95 (0.89, 1.02)	6	178633	18557	74
Overweight or obesity- (All nuts, [42], 2017)	0.97 (0.96, 0.99)	6	203591	28678	0

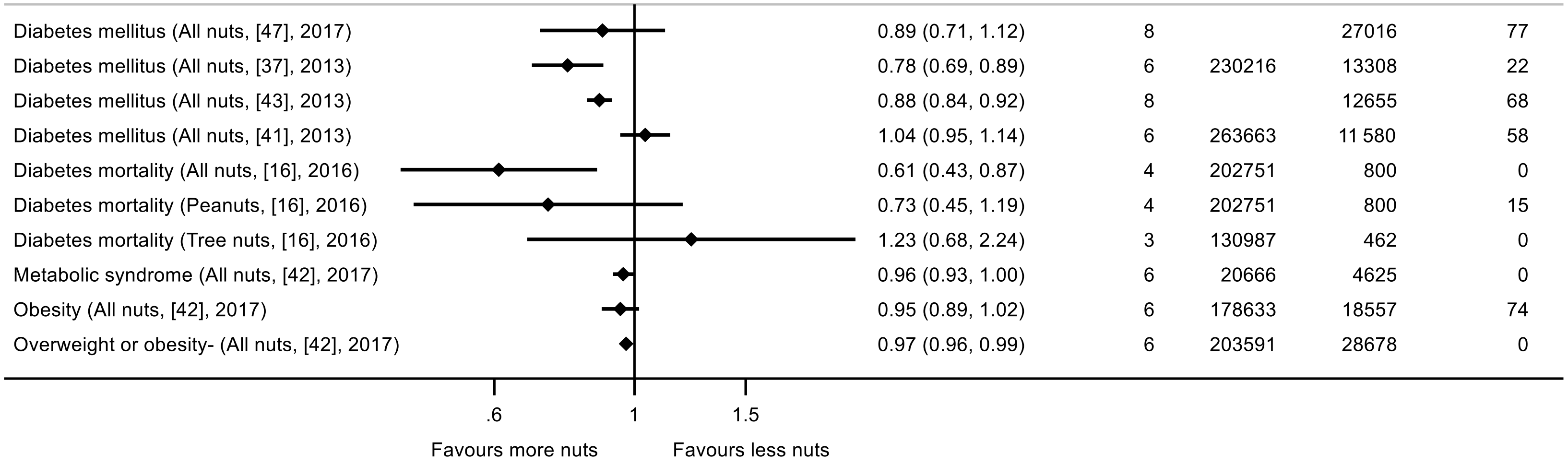

Supplementary figure 11: Summary of associations from meta-analyses between high compared to low consumption of nuts and risk of various other morbidities and mortalities. Reference number is listed in brackets and search year is listed within the parentheses. Number of studies, participants (n) and cases, as well as heterogeneity (I^2) is also listed in the right column.

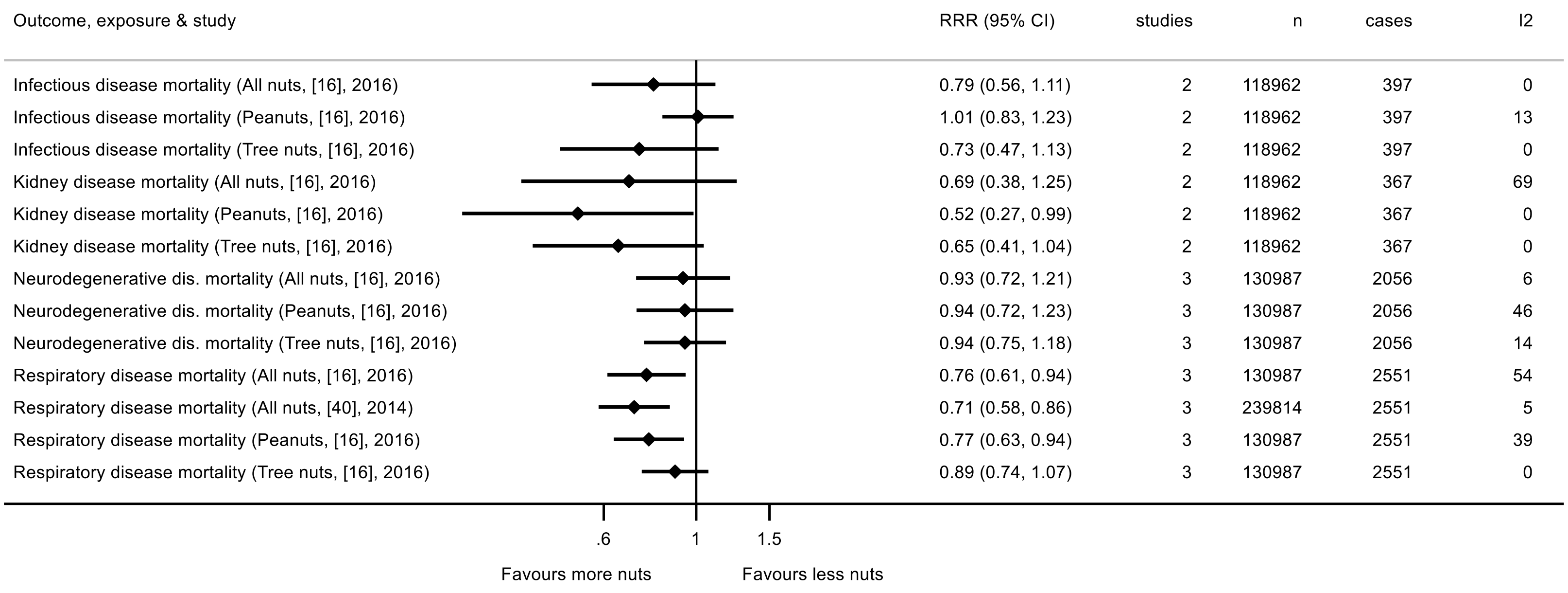

Supplementary figure 12: Summary of associations from meta-analyses between per serving of 28g consumption of nuts and risk of various other morbidities and mortalities. Reference number is listed in brackets and search year is listed within the parentheses. Number of studies, participants (n) and cases, as well as heterogeneity (I^2) is also listed in the right column.

Outcome, exposure & study	RRR (95% CI)	studies	n	cases	I2
Infectious disease mortality (All nuts, [16], 2016)	0.25 (0.07, 0.87)	2	118962	397	54
Infectious disease mortality (Peanuts, [16], 2016)	1.03 (0.81, 1.31)	2	118962	397	3
Infectious disease mortality (Tree nuts, [16], 2016)	0.64 (0.36, 1.13)	2	118962	397	0
Kidney disease mortality (All nuts, [16], 2016)	0.27 (0.04, 1.87)	2	118962	367	61
Kidney disease mortality (Peanuts, [16], 2016)	0.42 (0.24, 0.73)	2	118962	367	0
Kidney disease mortality (Tree nuts, [16], 2016)	0.66 (0.36, 1.21)	2	118962	367	0
Neurodegenerative dis. mortality (All nuts, [16], 2016)	0.65 (0.40, 1.07)	3	130987	2056	6
Neurodegenerative dis. mortality (Peanuts, [16], 2016)	0.92 (0.65, 1.31)	3	130987	2056	49
Neurodegenerative dis. mortality (Tree nuts, [16], 2016)	0.81 (0.58, 1.13)	3	130987	2056	26
Respiratory disease mortality (All nuts, [16], 2016)	0.48 (0.26, 0.89)	3	130987	2551	61
Respiratory disease mortality (Peanuts, [16], 2016)	0.69 (0.53, 0.90)	3	130987	2551	50
Respiratory disease mortality (Tree nuts, [16], 2016)	0.79 (0.62, 1.01)	3	130987	2551	0

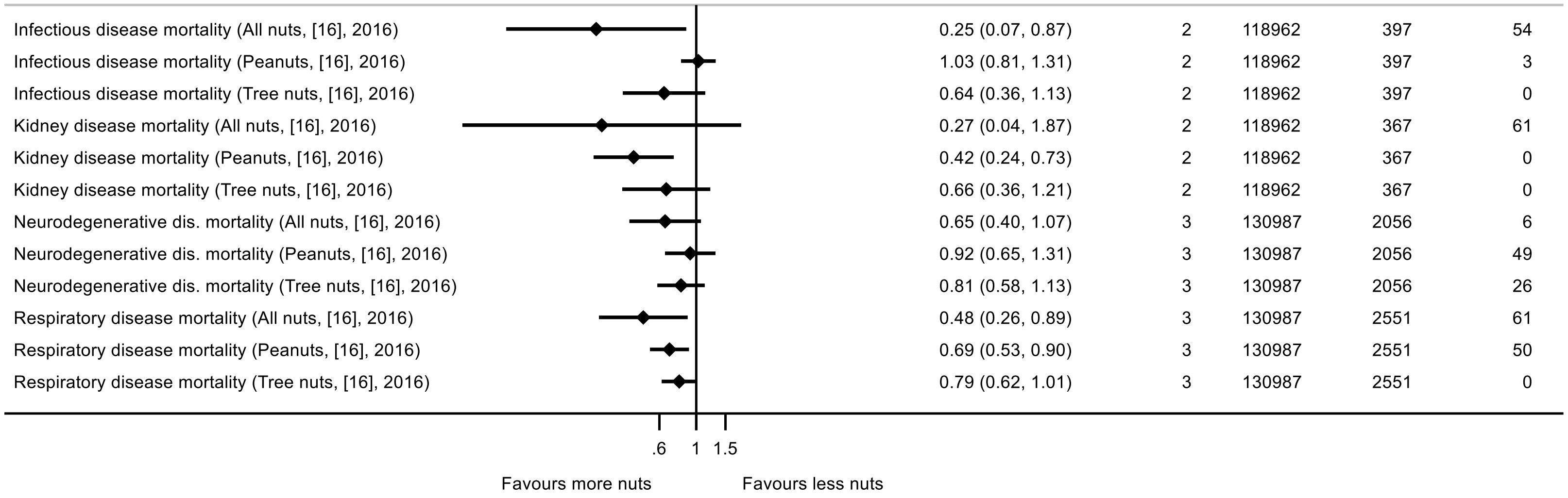

Supplementary figure 13: Summary of dose-response relationships from meta-analyses on associations between nut consumption of nuts and risk of cardiovascular diseases. The relationships are grouped by meta-analysis (labelled by first author). The meta-analysis by Aune et al. [16] is considered as most comprehensive. Reference numbers: Aune [16], Luo [43].

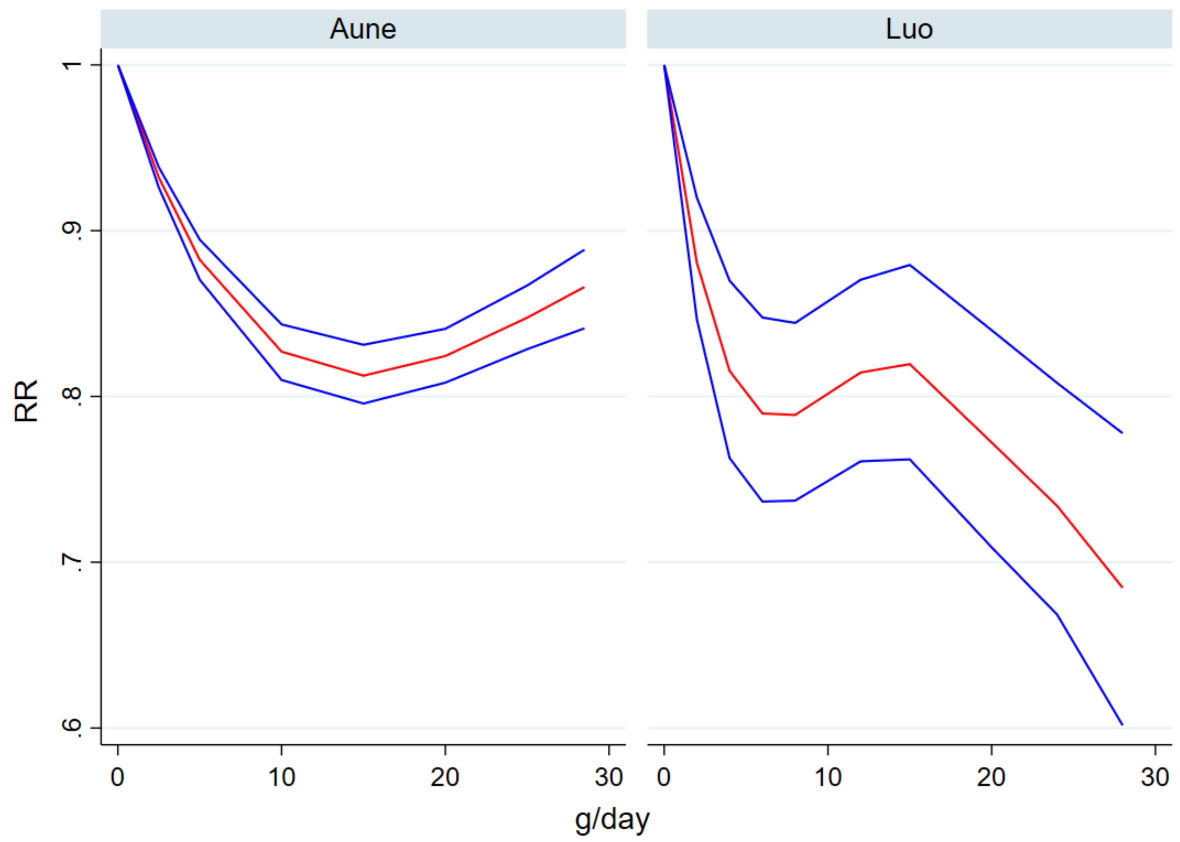

* The red lines indicate the relative risk associations estimate while the blue lines represent the confidence interval of the relative risks

Supplementary figure 14: Summary of dose-response relationships from meta-analyses on associations between nut consumption of nuts and risk of coronary heart/artery disease. The relationships are grouped by meta-analysis (labelled by first author). The meta-analysis by Aune et al. [16] is considered as most comprehensive. Reference numbers: Aune [16], Bechthold [39], Ma [44].

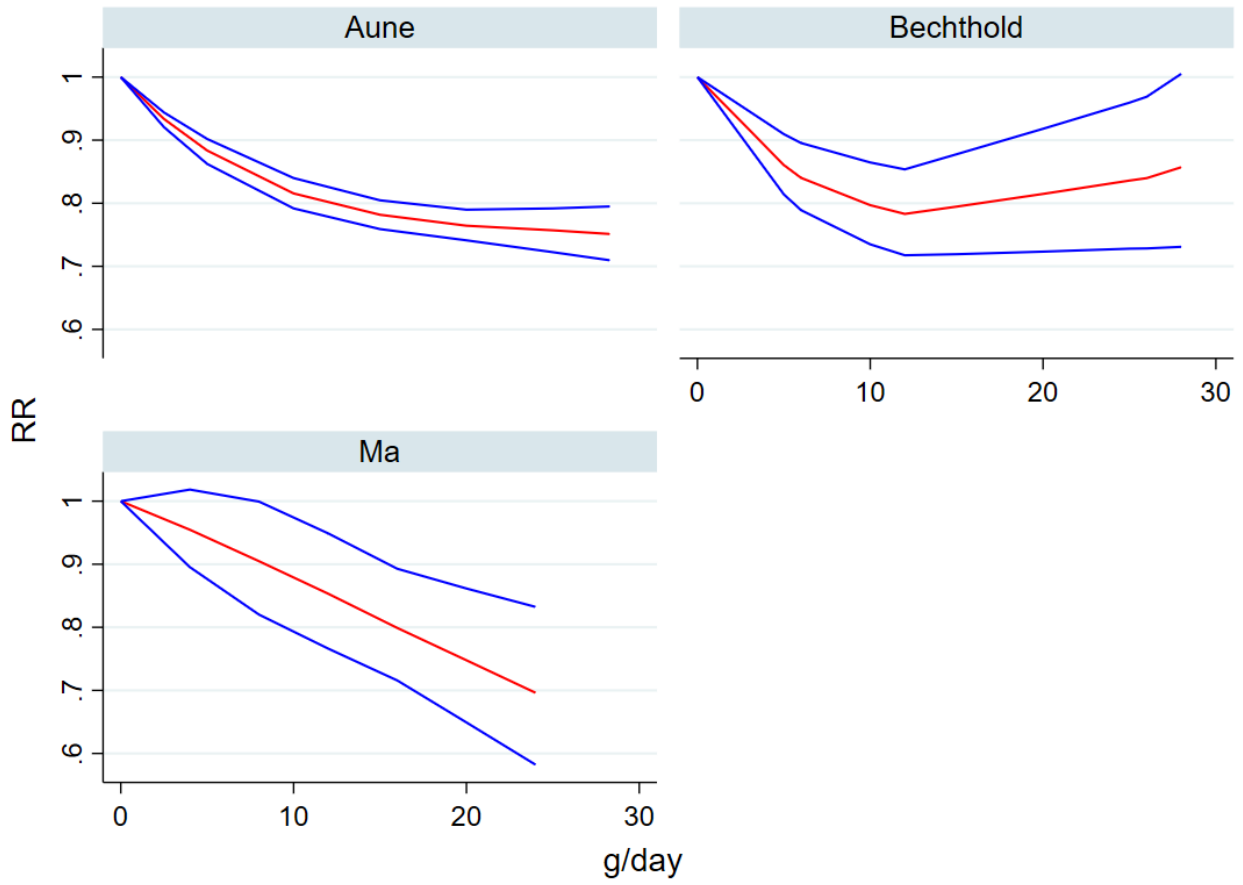

***** The red lines indicate the relative risk associations estimate while the blue lines represent the confidence interval of the relative risks

Supplementary figure 15: Summary of dose-response relationships from meta-analyses on associations between nut consumption of nuts and risk of stroke. The relationships are grouped by meta-analysis (labelled by first author). The meta-analysis by Aune et al. [16] is considered as most comprehensive. Reference numbers: Aune [16], Bechthold [39], Shao [48].

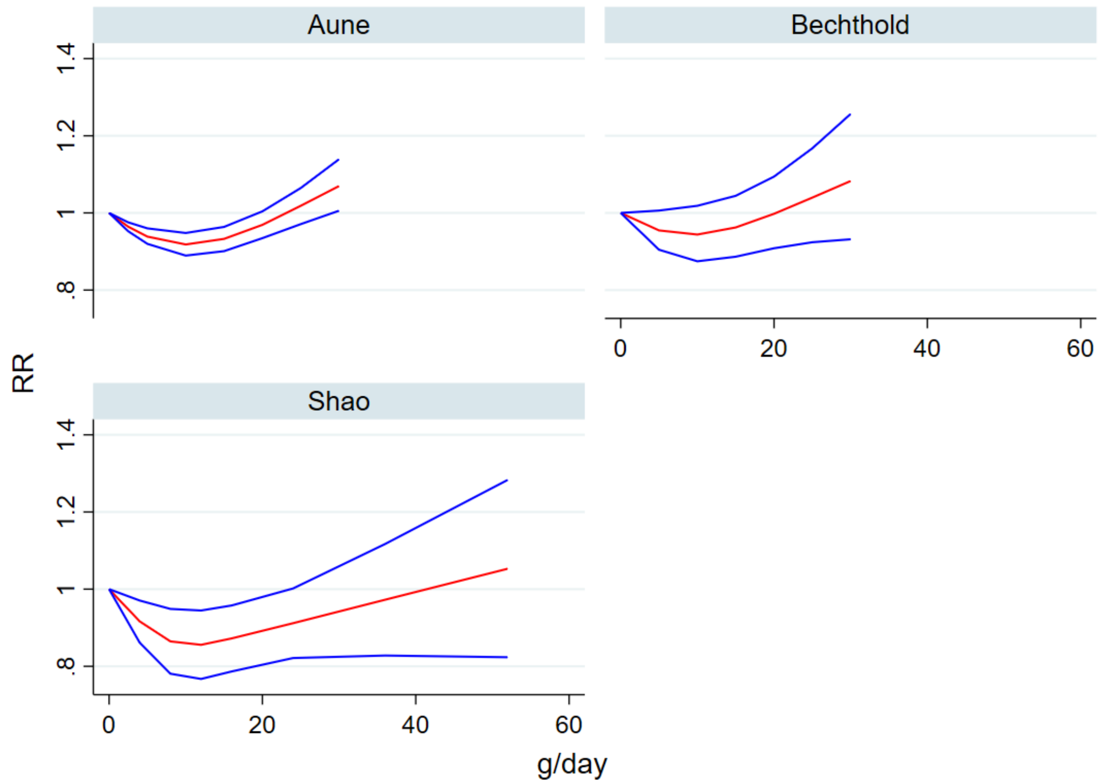

* The red lines indicate the relative risk associations estimate while the blue lines represent the confidence interval of the relative risks

Supplementary figure 16: Summary of dose-response relationships from meta-analyses on associations between nut consumption of nuts and risk of diabetes. The relationships are grouped by meta-analysis (labelled by first author). The meta-analysis by Becerra-Tomás, et al. [15] is considered as most comprehensive. Reference numbers: Becerra-Tomás [15], Luo [43], Schwingshackl [47].

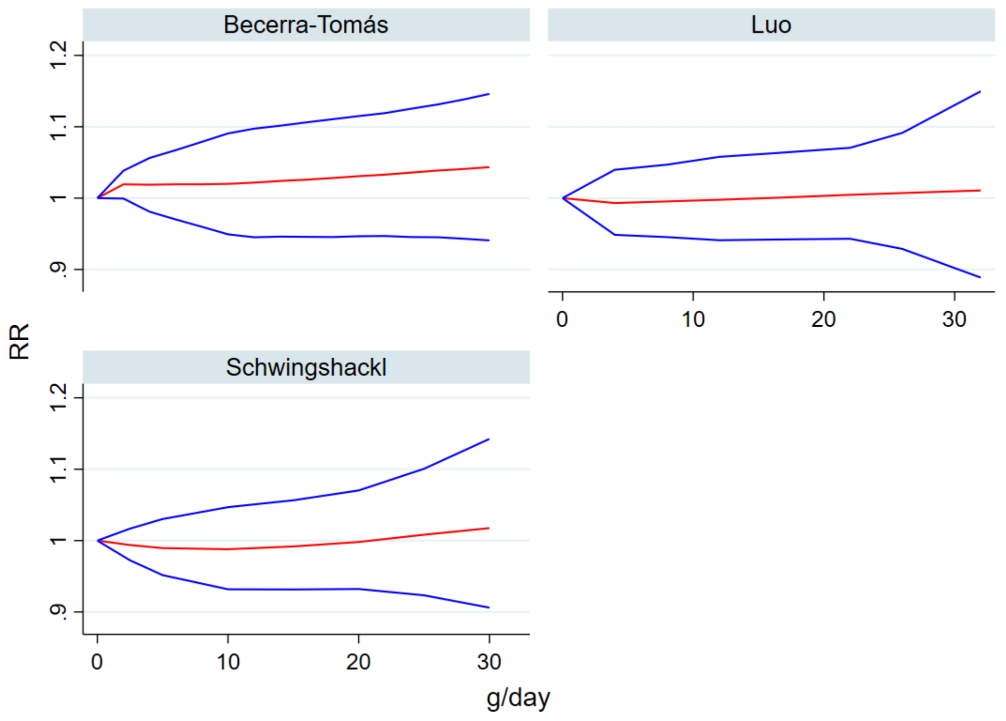

* The red lines indicate the relative risk associations estimate while the blue lines represent the confidence interval of the relative risks

Supplementary figure 17: Summary of dose-response relationships from meta-analyses on associations between nut consumption of nuts and risk of cancer. The relationships are grouped by meta-analysis (labelled by first author). The meta-analysis by Aune et al. [16], is considered as most reliable. Reference numbers: Aune [16].

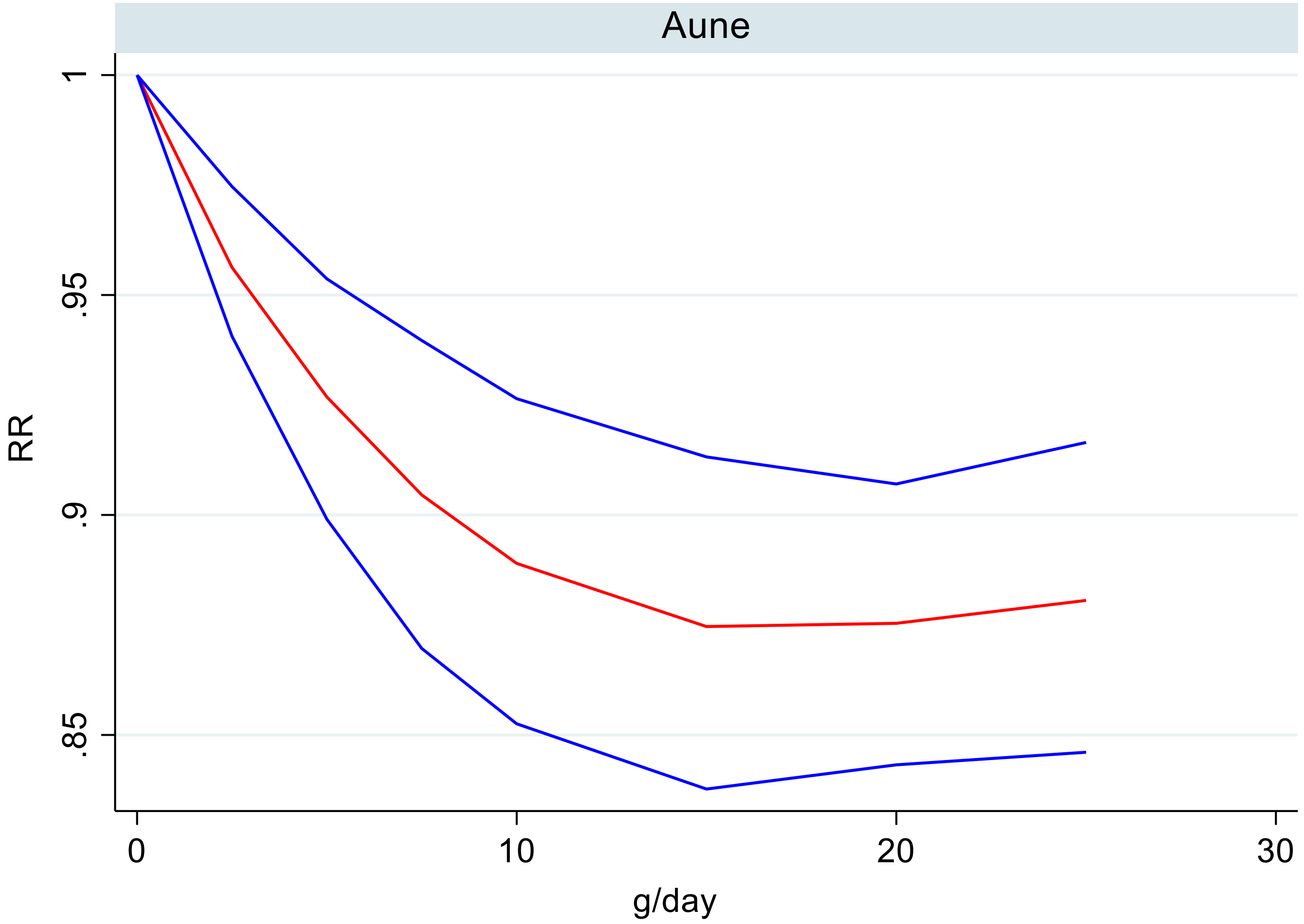

***** The red lines indicate the relative risk associations estimate while the blue lines represent the confidence interval of the relative risks

Supplementary figure 18: Summary of dose-response relationships from meta-analyses on associations between nut consumption of nuts and risk of mortality. The relationships are grouped by meta-analysis (labelled by first author). The meta-analysis by Aune et al. [16] is considered as most comprehensive. Reference numbers: Aune [16], Chen [13], Luo [43], Schwingshackl [46].

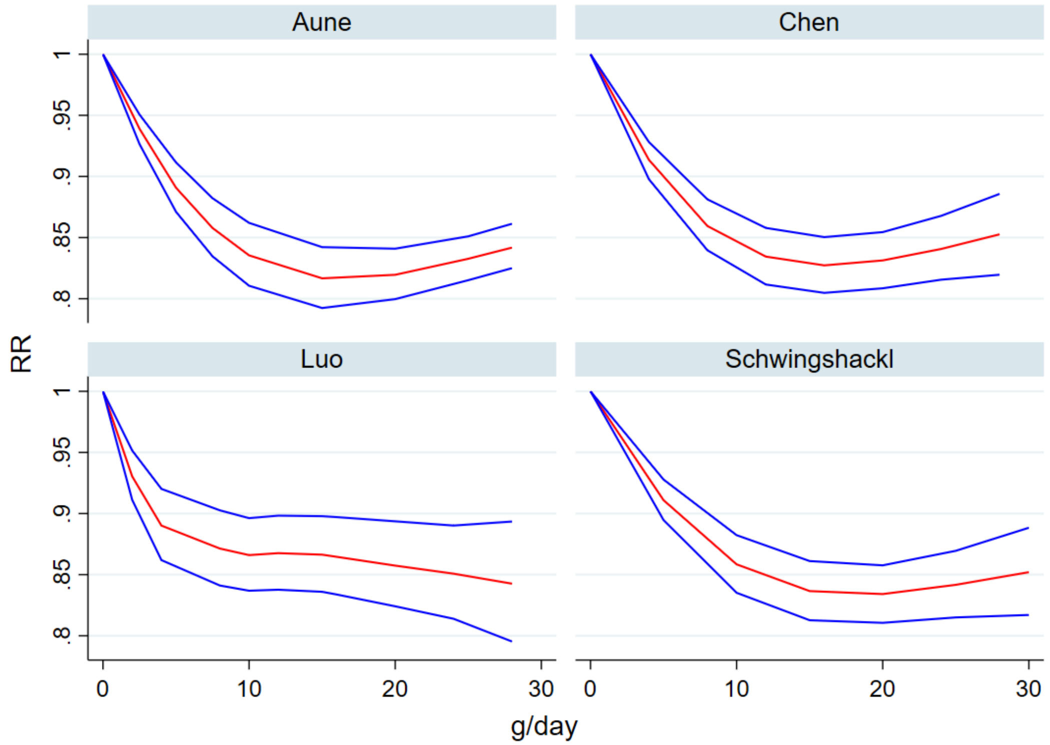

***** The red lines indicate the relative risk associations estimate while the blue lines represent the confidence interval of the relative risks

Supplementary text 1: Search documentation for literature search

Search documentation for literature search 27.5.2021

Overall, 1546 records were retrieved in 4 databases. After automatic deduplication in EndNote, 1009 records remained. After manual deduplication, ? records remained.

Epistemonikos:

The database seeks to import systematic reviews and higher level-syntheses from other databases, namely

- Cochrane Database of Systematic Reviews (CDSR) Last searched: May 27, 2021
- Pubmed Last searched: May 27, 2021
- EMBASE Last searched: May 25, 2021
- CINAHL (The Cumulative Index to Nursing and Allied Health Literature) Last searched: May 25, 2021
- PsycINFO Last searched: May 25, 2021
- LILACS (Literatura Latinoamericana y del Caribe en Ciencias de la Salud) Last searched: May 25, 2021
- Database of Abstracts of Reviews of Effects (DARE) Last searched: Aug 24, 2017 - No new records have been added to DARE after 2015
- The Campbell Collaboration online library Last searched: Nov 23, 2020
- JBI Database of Systematic Reviews and Implementation Reports Last searched: May 1, 2021
- EPPI-Centre Evidence Library Last searched: Mar 9, 2020

(read more)

The database does not contain a standardized vocabulary of subject headings, so the search was based on text words (with truncation used) in the title and abstract. There was only used one element in this search, i.e. nuts and seeds, as the database already filters for systematic reviews and other syntheses. The limitation to diet-related terms used in PubMed and Embase was omitted due to the lack of abstracts for many articles, which often would mention these terms.

The exported results include Epistemonikos' categories Systematic Review (521), Broad Synthesis (12) and Structured Summary (13). 546 results were retrieved and exported to EndNote.

Search strategy:

(title:(nut OR nuts OR almond* OR cashew* OR hazelnut* OR macadamia* OR paranut* OR peanut* OR pistachio* OR walnut* OR pecan* OR hickory OR seeds OR sesame OR "pine seed" OR "sunflower seed" OR chia OR "poppy seed" OR poppyseed* OR "hemp seed" OR hempseed* OR quinoa OR "pumpkin seed" OR flaxseed OR "flax seed") OR abstract:(nut OR nuts OR almond* OR cashew* OR hazelnut* OR macadamia* OR paranut* OR peanut* OR pistachio* OR walnut* OR pecan* OR hickory OR seeds OR sesame OR "pine seed" OR "sunflower seed" OR chia OR "poppy seed" OR poppyseed* OR "hemp seed" OR hempseed* OR quinoa OR "pumpkin seed" OR flaxseed OR "flax seed"))

PubMed (from inception to May 27 2021)

As the main element, a similar text word string for nuts and seeds was used as for Epistemonikos. The subject headings (MeSH) for nuts, seeds and individual species were added.

This element was combined with another set of text words and subject headings related to consumption, to limit the results to articles related to dietary use of nuts and seeds.

To limit the search to systematic reviews and meta-analyses, an adapted version of PubMed's subset for systematic reviews (systematic[sb]) and meta-analyses ("meta-analysis"[Publication Type]) was used. Several terms describing meta-analyses and systematic reviews have been added as text word search in title and abstract.

The search strategy was tested with 10 key articles, of which all were retrieved.

The search results (348) were exported to EndNote.

Search strategy:

("nuts"[MeSH Terms] OR "seeds"[MeSH Terms:noexp] OR "macadamia"[MeSH Terms] OR "bertholletia"[MeSH Terms] OR "carya"[MeSH Terms] OR "nut"[Title/Abstract] OR "nuts"[Title/Abstract] OR "almond*"[Title/Abstract] OR "cashew*"[Title/Abstract] OR "hazelnut*"[Title/Abstract] OR "macadamia*"[Title/Abstract] OR "paranut*"[Title/Abstract] OR "peanut*"[Title/Abstract] OR "pistachio*"[Title/Abstract] OR "walnut*"[Title/Abstract] OR "pecan*"[Title/Abstract] OR "hickory"[Title/Abstract] OR "seeds"[Title/Abstract] OR "sesame"[Title/Abstract] OR "pine seed"[Title/Abstract] OR "sunflower seed"[Title/Abstract] OR "chia"[Title/Abstract] OR "poppy seed"[Title/Abstract] OR "poppyseed*"[Title/Abstract] OR "hemp seed"[Title/Abstract] OR "hempseed*"[Title/Abstract] OR "quinoa"[Title/Abstract] OR "pumpkin seed"[Title/Abstract] OR "flaxseed*"[Title/Abstract] OR "flax seed"[Title/Abstract]) AND ("diet"[MeSH Terms] OR "eating"[MeSH Terms] OR "intake*"[Title/Abstract] OR "consum*"[Title/Abstract] OR "eating"[Title/Abstract] OR "eat"[Title/Abstract] OR "eats"[Title/Abstract] OR "diet*"[Title/Abstract]) AND ((((("systematic review"[Title] OR "systematic literature review"[Title] OR "systematic scoping review"[Title] OR "systematic narrative review"[Title] OR "systematic qualitative review"[Title] OR "systematic evidence review"[Title] OR "systematic quantitative review"[Title] OR "systematic meta review"[Title] OR "systematic critical review"[Title] OR "systematic mixed studies review"[Title] OR "systematic mapping review"[Title] OR "systematic cochrane review"[Title] OR "systematic search and review"[Title] OR "systematic integrative review"[Title]) NOT "comment"[Publication Type]) NOT ("protocol"[Title] OR "protocols"[Title])) NOT "MEDLINE"[Filter]) OR ("cochrane database syst rev"[Journal] AND "review"[Publication Type]) OR "systematic review"[Publication Type] OR "meta-analysis"[Publication Type] OR "meta-analysis as topic"[MeSH Terms] OR "meta analy*"[Title/Abstract] OR "meta analy*"[Title/Abstract] OR "metanaly*"[Title/Abstract] OR "metaanaly*"[Title/Abstract] OR "met analy*"[Title/Abstract] OR "integrative review*"[Title/Abstract] OR "integrative overview*"[Title/Abstract] OR "collaborative review*"[Title/Abstract] OR "collaborative overview*"[Title/Abstract] OR "systematic review*"[Title/Abstract] OR "systematic overview*"[Title/Abstract])

Cochrane Database of Systematic Reviews (Issue 5 of 12, May 2021)

A similar text word search string for nuts and seeds was used as for the other databases. Text word search was performed for the fields title, abstract and keywords. Additionally, MeSH terms for nuts and seeds were used.

29 systematic reviews were retrieved and exported to EndNote.

Search strategy:

#1 (nut OR nuts OR almond* OR cashew* OR hazelnut* OR macadamia* OR peanut* OR pistachio* OR walnut* OR pecan* OR hickory OR seeds OR sesame OR "pine seed" OR "sunflower seed" OR chia OR "poppy seed" OR poppyseed* OR "hemp seed" OR quinoa OR "pumpkin seed" OR flaxseed* OR "flax seed"):ti,ab,kw
#2 MeSH descriptor: [Seeds] explode all trees
#3 MeSH descriptor: [Nuts] explode all trees
#4 MeSH descriptor: [Macadamia] explode all trees
#5 MeSH descriptor: [Bertholletia] explode all trees
#6 MeSH descriptor: [Carya] explode all trees
#7 #1 OR #2 OR #3 OR #4 OR #5 OR #6

Embase:

The main element included the same text word string for nuts and seeds as for Epistemonikos, PubMed and Cochrane. Subject headings (Emtree) for nuts, seeds and individual species were added.

This element was combined with another set of text words and subject headings related to consumption, to limit the results to articles related to dietary use of nuts and seeds.

All text word searches were applied to the search fields title, abstract and keywords.

To limit the search to systematic reviews and meta-analyses, the filter used for the search in PubMed was translated to Embase (Ovid).

The search results (623) were exported to EndNote.

Search strategy:

1 nut/ or almond/ or brazil nut/ or cashew nut/ or hazelnut/ or pecan/ or pistachio nut/ or walnut/ or peanut/ or macadamia/ or sesame/ or exp pseudocereal/ or exp oilseed/
2 (nut or nuts or almond* or cashew* or hazelnut* or macadamia* or peanut* or pistachio* or walnut* or pecan* or hickory or seeds or sesame or "pine seed" or "sunflower seed" or chia or "poppy seed" or poppyseed* or "hemp seed" or quinoa or "pumpkin seed" or flaxseed* or "flax seed").ti,ab,kw.
3 1 or 2
4 exp food intake/
5 (intake* or consum* or eat* or diet*).ti,ab,kw.
6 4 or 5
7 "systematic review"/ or meta analysis/ or "meta analysis (topic)"/ or "systematic review (topic)"/
8 ((systematic* adj3 (review* or overview*)) or (integrative adj3 (review* or overview*)) or (collaborative adj3 (review* or overview*)) or (met analy* or metanaly* or meta-analy* or metaanaly* or systematic review*)).ti,ab,kw.
9 7 or 8
10 3 and 6 and 9
